# Model reduction in mathematical pharmacology: Integration, reduction and linking of PBPK and systems biology models

**DOI:** 10.1007/s10928-018-9584-y

**Published:** 2018-03-26

**Authors:** Thomas J. Snowden, Piet H. van der Graaf, Marcus J. Tindall

**Affiliations:** 1 Department of Mathematics and Statistics, University of Reading, Reading, RG6 6AX UK; 2 Certara QSP, University of Kent Innovation Centre, Canterbury, CT2 7FG UK; 3 Leiden Academic Centre for Drug Research, Universiteit Leiden, 2333 CC Leiden, The Netherlands; 4 The Institute for Cardiovascular and Metabolic Research (ICMR), University of Reading, Reading, RG6 6UR UK

**Keywords:** Mathematical pharmacology, Model reduction, Systems pharmacology, PBPK

## Abstract

In this paper we present a framework for the reduction and linking of physiologically based pharmacokinetic (PBPK) models with models of systems biology to describe the effects of drug administration across multiple scales. To address the issue of model complexity, we propose the reduction of each type of model separately prior to being linked. We highlight the use of balanced truncation in reducing the linear components of PBPK models, whilst proper lumping is shown to be efficient in reducing typically nonlinear systems biology type models. The overall methodology is demonstrated via two example systems; a model of bacterial chemotactic signalling in Escherichia coli and a model of extracellular regulatory kinase activation mediated via the extracellular growth factor and nerve growth factor receptor pathways. Each system is tested under the simulated administration of three hypothetical compounds; a strong base, a weak base, and an acid, mirroring the parameterisation of pindolol, midazolam, and thiopental, respectively. Our method can produce up to an 80% decrease in simulation time, allowing substantial speed-up for computationally intensive applications including parameter fitting or agent based modelling. The approach provides a straightforward means to construct simplified Quantitative Systems Pharmacology models that still provide significant insight into the mechanisms of drug action. Such a framework can potentially bridge pre-clinical and clinical modelling - providing an intermediate level of model granularity between classical, empirical approaches and mechanistic systems describing the molecular scale.

**Electronic supplementary material:**

The online version of this article (doi:10.1007/s10928-018-9584-y) contains supplementary material, which is available to authorized users.

## Introduction

Within the past decade quantitative systems pharmacology (QSP) has emerged as a novel discipline proposing the use of integrated, multidisciplinary models that bridge the gap between the biological insight of modelling target scale effects of drug action systemically with pharmacokinetic/pharmacodynamic (PKPD) modelling approaches traditionally found in the field of clinical pharmacology [49, 59, 61, 65]. The aim of such an approach is to obtain mechanistic models of drug action that enable the prediction of drug dose-exposure, efficacy and potential side effects for a given subject and dose a priori. Some researchers [60] see the approach as providing a partial remedy to the current issues of candidate attrition troubling the pharmaceutical industry and a stepping stone towards an ultimate goal of personalised medicine.

The core principle of QSP is the bringing together of data and knowledge from basic biological research and the multiple stages of drug development into a single multiscale quantitative modelling framework describing drug action. At its simplest this means the integration of cell level signalling detail (e.g. protein-protein interaction networks) with multiscale models that span the effect of drug binding at the molecular level up to the whole-body effects of drug administration, absorption and clearance. The approach has a number of potential benefits compared with empirical compartmental modelling approaches commonly used to describe population PKPD. Firstly, it offers an integrated modelling approach to drug development bringing together experimental results from preclinical, animal and clinical studies into a unified quantitative model of drug action, enabling a more mechanistic approach to the study of translation. Secondly, it can provide greater insight into the mechanisms of action underlying drug efficacy and toxicity, with mathematical analysis of molecular scale signalling models potentially enabling the study and prediction of emergent cell scale network phenomena that may not have been predictable via traditional PKPD approaches. Thirdly, it can yield better mechanistic understanding of the possible causes of between-patient variability. Finally, it enables the integration of data from previous drug candidates of similar classes (failures and successes) that act on the same or related pathways. In doing so, it can provide a more nuanced framework for studying the causes of drug candidate failure and how they can be avoided in future.

Whilst QSP offers an approach for integrating knowledge across multiple scales in the prediction of drug efficacy, it raises a number of mathematical challenges [61]. These include developing the tools to create and validate multiscale models potentially ranging from the genetic to population level, surmounting the issues of practicability associated with highly complex, nonlinear models of biochemical reaction cascades, and addressing the general difficulties associated with meaningfully combining interdisciplinary data. In this paper we outline a framework for the creation of QSP models through the reduction and linkage of physiologically based pharmacokinetic (PBPK) models with reduced systems biology type models describing the biochemical activity of a drug at the cellular and intracellular scales.

Pharmacological modelling of drug disposition now commonly employs PBPK at multiple stages of research and development [17, 18, 32]. Such systems describe the movement of a drug throughout compartments corresponding to realistic tissues that span the entire body. PBPK models differ from the classical compartmental approaches to pharmacokinetic modelling in that they incorporate parameters informed via physiological and biological knowledge of the body in order to more mechanistically, as opposed to empirically, describe pharmacokinetic activity. Due to the higher dimensionality of these models, and associated issues of parameterisation to which such a modelling approach can lead, PBPK remains most commonly applied within the context of drug discovery and for the scaling of in vitro or animal studies of drug disposition. At clinical stages of drug development, PBPK can still find use in drug-drug interaction or pediatric studies, but where clinical trials are performed in order to initially fit models to in vivo experimental data, simpler and more empirical approaches to PK modelling still tend to be preferred. In part, this is due to the fact that PBPK models are often found to be structurally unidentifiable [64], greatly hindering their practical use in the context of clinical trial data. Partly in order to improve such properties of identifiability and to enable parameter fitting, publications concerning the reduction of PBPK models, have begun to emerge [57].

As an approach to mathematically modelling biological systems, systems biology differs philosophically from preceding approaches in that it attempts to describe cells and their signalling systems holistically [19, 20]. This enables the creation of models that incorporate explanatory power of underlying biological mechanisms at the cost of significant model complexity. This is typically achieved by describing systems at a molecular level of detail and showing how their interaction can produce larger scale phenomena of interest. Such approaches have gained some traction within a pharmacological setting due to their capacity to describe a drug’s mechanism of action physiologically.

**Fig. 1:**
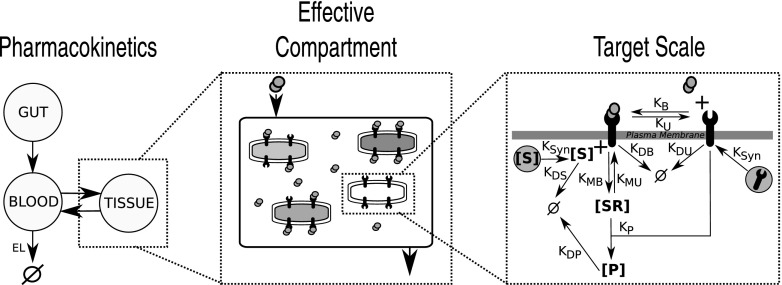
Multiple scales of drug action. Our approach seeks to bring together models from across multiple scales of drug action into a single framework. Here the whole body scale is represented by a model of pharmacokinetics, where the effective compartment (in this case the tissue) comprises a model of diffusing drug molecules. The molecular or target scale incorporates a description of drug-receptor binding and the underlying signalling cascade dynamics (the systems biology scale). The example given here applies to G protein-coupled receptor type drugs targets, but the approach is valid more generally

By combining PBPK and systems biology modelling approaches, it is hence hypothetically possible to model drug disposition and dose-response mechanistically as opposed to the traditionally empirical description provided by classical PKPD. Rather than fitting an abstract model to the clinically observed data, the aim here is to produce a model that describes in detail the main physiological processes at work. This leads to a multiscale modelling approach spanning the scales and processes depicted, for example, in Fig. 1. Here, a PBPK model describes the absorption, distribution and clearance of the drug through the body, whilst in a given effective compartment a Systems Biology type model describes how the effective concentration of the drug elicits a response at the target scale. Figure 1 shows a receptor type target of drug binding, but the general idea is valid across many target types.

Despite their potential usefulness, however, models attempting to span the scales of both PBPK and systems biology will typically be significantly too complex to be of practical use in a clinical setting. This complexity can be seen to stem from a number of common mathematical properties. For instance, such models will often possess a very large number of modelled species, concentrations and reactions. Such systems are often modelled using the theory of deterministic ordinary differential equations (ODEs) and as such can comprise tens to hundreds of state-variables. Such models thus often come with associated issues of high simulation time and numerical error. Models also frequently describe a wide range of kinetic rates observed across the multiple scales of drug action and as a result, portions of the system are likely to evolve on greatly different time-scales resulting in model stiffness. Such models are also usually nonlinear, which can prohibit a number of analytical approaches. Finally, due to the scope of these systems, it can simply prove too difficult to readily intuit or understand their biological implications. Often the parameter space is simply too large to convincingly explore or understand what variation in the parameterisation may mean.

One methodology for tackling a number of these issues, in an effort to bring understanding of the role of processes within and across scales, is model reduction. Model reduction here refers to any method designed to construct a simplified formulation of a model with which some set of the original dynamical behaviour can be satisfactorily approximated and within which some degree of predictive power is retained. A wide variety of such methods exist in the literature [2, 34, 47] and they have commonly been employed for alleviating issues of complexity in other fields of modelling (for example chemical engineering [34], control theory [44], and weather prediction [26]). Within the context of integrative QSP, model reduction can potentially be applied at multiple levels. In this paper we evaluate two particular uses in this context; firstly, model reduction can be used to yield a simplified description of the pharmacokinetic disposition of a drug that retains a physiological basis. Secondly, it be can be used to produce a reduced description of the biochemical activity of the drug at the target scale through the simplification of systems biology type models.

The simplification of PBPK models has been relatively well explored in the literature through the use of linear, proper lumping [4, 10, 12, 33, 37]. The aim is typically to reduce the more complex models of PBPK to the point where they can be fit against clinical trial data as in the case of traditional compartmental PK models [57]. Proper lumping has also seen application in the broader contexts of Systems Biology [7, 9, 21, 50, 51] and Systems Pharmacology [14] type models. Other model reduction approaches including time-scale exploitation [6, 13, 22, 23, 36, 39, 43, 52–54], sensitivity analysis [3, 8, 28, 29, 45], optimisation based approaches [1, 30, 38, 55] and balanced truncation [15, 27, 48] have also seen published application within a systems biology setting. Here, we focus specifically on two of these methods - proper lumping and balanced truncation. Proper lumping seeks to reduce a system by modelling the dynamical behaviour of subsets of the original state-variables en masse as opposed to individually. Meanwhile balanced truncation transforms the model’s state-variables into a form where those portions of the network least responsible for some input–output type relationship of interest can be easily removed.

The ideal scope and complexity of model depends necessarily on the specific questions that we are seeking to address, the level of approximation we are willing to accommodate, the prior knowledge we have at hand, and the actual data available. In practice, modelling often boils down to a balance between these factors. When constructing models from the ground up, these considerations often have to be repeatedly assessed—and discussions around how to achieve this do exist in the literature [63]. One of the key advantages of model reduction, however, is that we can instead start by constructing a complex model based upon the full extent of the literature available, and then use automated methods to reduce it down to a scale appropriate to its intended application. It is this approach that we seek to leverage throughout this paper—by first assembling a holistic, physiological description of a drug’s mechanism of action alongside its pharmacokinetic disposition, we then aim to apply model reduction to the individual components of this model in order to automatically extract a practical and usable system that retains an explicit link back to this physiological scale.

The literature concerning the other step of linking of PK modelling efforts with those of systems biology is less developed than that of model reduction. Krippendorff et al. [24] have demonstrated a simple linking procedure whereby a mass-action model is built integrating the whole-body and cellular scales. Both normal and diseased cells exist in a well-stirred compartment of the PK model each with receptors able to bind to the drug. They demonstrate that this approach can potentially be used to study how such differences in receptor affinity affects the clinical response of drugs with the same proposed mechanism of action. It is this approach to model linkage that we employ throughout our work.

Given the context outlined above, our paper demonstrates how methods of model reduction and model linkage can be brought together under a single framework in order to yield simplified Systems Pharmacology or enhanced pharmacodynamics models [16]. The developed methodology is applicable to models formulated using deterministic nonlinear and linear ODEs. For a given drug, the framework presented here starts with a model of PBPK and a relevant systems biology model describing the drug’s hypothesised mechanism of action at the target scale. The approach applies differing methods of model reduction to individual components of the network based on their suitability, and then recombines the reduced components to finally obtain a simplified system. This work is related to our previous paper [46] which developed a combined model reduction algorithm that sequentially applied multiple methods of reduction in order to obtain highly accurate reduced systems.The overall method, however, does not sequentially apply reduction methods, but instead seeks to decompose the overall network into linear and nonlinear sub-modules and then reduce them independently using the most appropriate method for each. By focusing on the maintenance of input–output relations for each sub-module throughout its reduction, we allow the overall model to remain highly accurate when recombined. As is demonstrated, these models continue to provide an accurate description of drug action across multiple scales whilst also having been reduced to a significantly more tractable size. Additionally, our approach in the reduction of PBPK models is differentiated from those previously published in that we seek to apply balanced truncation, as opposed to proper lumping, as a means of simplification. The framework is demonstrated using a generalised PBPK model and systems biology models of differing complexity: an 11 dimensional model of bacterial chemotactic signalling in *Escherichia coli* [56] and a 99 dimensional model of extracellular signal-regulated kinase (ERK) phosphorylation mediated via the epidermal growth factor (EGF) and nerve growth factor (NGF) receptor pathways [42].

## Methodology

There are several possible approaches for the creation of a reduced model spanning the multiple scales of drug activity. This paper assumes that one begins with, or is able to develop, models of the drug’s pharmacokinetic behaviour and the target scale activity describing its proposed mechanism of action. The aim is then to create a single system encapsulating the dynamics of both models whilst also being simple enough to be practically usable within a clinical setting.

Given this starting point, the overall aim is comprised of two major steps; model reduction and model linkage. There are two possible routes to achieving this:

1. Linking the models together initially and reducing the entire linked system under a single approach to obtain the reduced linked system; or
2. Initially seeking to separate or decompose the constituent models into sub-modules with related properties. Then, seek to reduce these modules in isolation, using the most appropriate method of reduction for each model component. Finally we can then link the reduced modules together, again yielding a reduced linked system.

Whilst the first approach may allow a simpler mathematical implementation by only requiring a single model reduction approach, this fact is also its main disadvantage in that the method employed must be valid for all aspects of the model and is unlikely to be optimal for any of them. Instead this paper outlines the use of the second approach, which allows us to tailor the methods of model reduction to specific model components and hence obtain better, more accurate overall reduced systems. The reasons for wanting to achieve this are twofold.

**Fig. 2:**
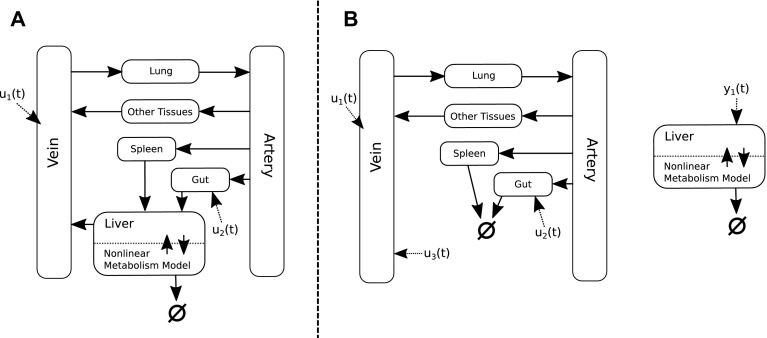
Example depiction of a linear/nonlinear decomposition of a PBPK model. **a** Depicts an example schematic of a PBPK model, which includes some nonlinear description of metabolism occurring in the liver compartment. Here inputs $u_1(t)$ and $u_2(t)$ refer to the time-courses of IV doses and oral doses respectively. **b** Shows how the model can be decomposed into linear and nonlinear components. $y_1(t)$ represents an output of the linear portion of the model which feeds into the liver compartment, and $u_3(t)$ is an input into the model, representing the distribution of the drug from the liver to the venous compartment

Firstly, although models of PBPK are often nonlinear, they can often be decomposed into linear and nonlinear components. The linear components would typically include a description of the inter-compartmental distribution of the drug throughout various tissues of the system, whilst the nonlinear components tend to include intra-compartmental components (covering examples such as Michaelis-Menten like metabolism in the liver, saturable plasma protein binding, and interaction with transporters or target-mediated drug disposition within some effective compartment). These nonlinear components can often be ‘disintegrated’ from the linear model, and intead represented by arbitrary input and output terms within the linear network. Similar decomposition approaches have previously been described in the area of modular response theory and applied to systems biology type models to good effect [5, 27]. An example of such a decomposition is presented in Fig. 2. Given such a decomposition it is possible to employ more efficient, linear methods of model reduction that globally preserve the input–output relationship of the system (such as balanced truncation) for the reduction of the linear portion of the network.

Secondly, the timescales of PBPK models are typically significantly slower than those of biochemical reaction network models. As a result, reduction of the pre-linked system will often remove much more of the detail from the systems biology model which may not be ideal in the case where mechanistic explanatory power at this scale is intended to be retained.

**Fig. 3:**
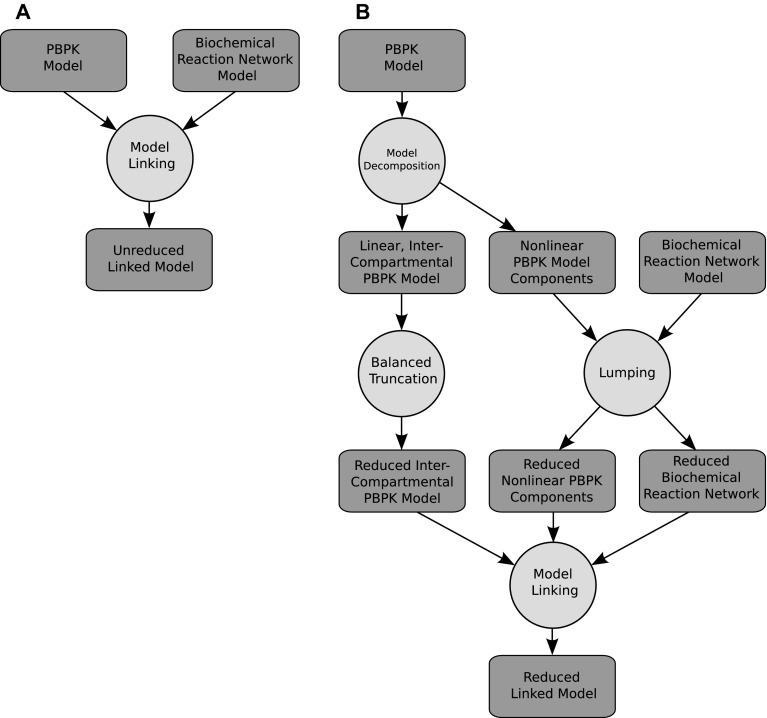
Proposed schematics for the reduction and linking of PBPK and systems biology modelling approaches. **a** Depicts a schematic for the creation of what is here referred to as the ‘unreduced linked model’. **b** Depicts the recommended schematic for the creation of what is here referred to as the ‘reduced linked model’. Circles indicate a methodology to be applied whilst the rounded rectangles indicate the type of model thereby produced

The overall approach for achieving a reduced linked system proposed here is depicted in Fig. 3b. The method begins with the unreduced PBPK and systems biology intracellular biochemical reaction network models. To reduce the PBPK model we then decompose it into its linear and nonlinear components as previously described. Inputs and outputs are then defined based upon this decomposition, the specific modelled phenomena of interest, and to represent how the PK drives the modelled systems biology processes. Next the linear components of the model are reduced via balanced truncation under the defined input–output terms. All nonlinear components, typically including the biochemical reaction network, are then reduced via proper lumping. Finally, the reduced model components are then linked.

Crucially, points of coupling between models or model components, as well as any imposed model linkages, can be addressed by defining the outputs of one model or component to represent the inputs of those it affects; an example of which is given in Fig. 2. The reduced models are then constructed so as to be able to maintain this input–output behaviour, thus guaranteeing the relative accuracy of the reduced ensemble of models when recombined. Once constructed, the performance of such reduced linked systems can be compared to the ‘unreduced linked system’ as depicted in Fig. 3a.

Given this overall framework, the remainder of this methodology section provides more specific, mathematical detail on the overall problem and a basic account of the reduction methods – proper lumping and balanced truncation.

### Model reduction and approximation error

Throughout this paper we seek to reduce both PBPK and Systems Biology type models. In both cases such physical systems are generally described by systems of coupled, nonlinear ODEs. For our purposes here, we additionally formulate these systems as initial value problems and express them via a control affine, state-space representation such that

1a $$\begin{aligned} \dot{{\varvec{x}}}(t)= \,& {} \varvec{f}({\varvec{x}}(t)) + \sum _{i=1}^l\varvec{g}_i({\varvec{x}}(t)) u_i(t), \end{aligned}$$

1b $$\begin{aligned} {\varvec{y}} (t)= \,& {} \varvec{h}({\varvec{x}} (t)), \end{aligned}$$

with initial conditions ${\varvec{x}}(0)={\varvec{x}}_0$ and where the over-dot represents the time-derivative (such that, $\dot{{\varvec{x}}} = \frac{{\mathrm{d}}{\varvec{x}}}{{\mathrm{d}}t}$). Here ${\varvec{x}}(t)\in {\mathbb{R}}^n$ represents the model state-variables (e.g. the time-varying concentrations of the modelled species or the compartmental drug concentrations), ${\varvec{u}}(t)\in {\mathbb{R}}^l$ (such that $u_i(t)\in {\varvec{u}}(t)$) represent the input variables (e.g. the initial, repeated or continuous doses that are mapped to the relevant pharmacokinetic compartments), and ${\varvec{y}}\in {\mathbb{R}}^p$ represent the output variables. Here, $\varvec{f}({\varvec{x}}(t))$ is the set of functions describing the dynamical interaction between the state-variables, each set of functions $\varvec{g}_i({\varvec{x}}(t))$ describes how the inputs feed into the state-variable dynamics and $\varvec{h}({\varvec{x}} (t))$ describes the combinations of the state-variables corresponding to each of the outputs. Note that in the linear case, common in the study of pharmacokinetics, the original system (1) can be expressed in the form

2a $$\begin{aligned} \dot{{\varvec{x}}}(t)= \,& {} A{\varvec{x}}(t) + B{\varvec{u}}(t), \end{aligned}$$

2b $$\begin{aligned} {\varvec{y}}(t)= \,& {} C{\varvec{x}}(t), \end{aligned}$$

where *A*, *B*, and *C* are linear operators, such that $A:\,{\mathbb{R}}^n\rightarrow {\mathbb{R}}^n$, $B:\,{\mathbb{R}}^l\rightarrow {\mathbb{R}}^n$ and $C:\,{\mathbb{R}}^n\rightarrow {\mathbb{R}}^r$.

Whilst model input and output can be fairly abstract concepts, within a pharmacological context they can be reasonably concretely understood. In the case of a PBPK type model the input could, for example, describe the dosing regimen administered, whilst the output might correspond to the concentration of the compound in some subset of the modelled compartments. In the case of a systems biology type model describing a receptor signalling pathway, the input might represent the time-varying, extracellular concentration of a specific ligand, whilst the output may represent the concentration of a specific intracellular protein associated with a cellular response of interest.

Given such a formulation, we then seek a reduced model of the form

3a $$\begin{aligned} \dot{\tilde{{\varvec{x}}}}(t)= \,& {} \tilde{\varvec{f}}(\tilde{{\varvec{x}}}(t))+ \sum _{i=1}^l \tilde{\varvec{g}}_i(\tilde{{\varvec{x}}}(t))u_i(t), \end{aligned}$$

3b $$\begin{aligned} \bar{{\varvec{y}}}(t)= \,& {} \tilde{\varvec{h}}(\tilde{{\varvec{x}}}(t)), \end{aligned}$$

where $\sim$ denotes an approximation of reduced dimension for the equivalent, original term in Eq. (1). Additionally, $\bar{{\varvec{y}}}(t)\in {\mathbb{R}}^p$ represents an approximation of the original output ${\varvec{y}}$ calculated from the values of the reduced state-variables $\tilde{{\varvec{x}}}$.

The accuracy of the reduced models can be quantified by a number of approaches, often dependent upon the specific aims of reduction. The most common approaches are based upon measures of the instantaneous error between the outputs of the two systems, $\left| {\varvec{y}}(t) - \bar{{\varvec{y}}}(t)\right|$. Throughout this paper we use a measure of maximal relative error $\varepsilon$, such that

4 $$\begin{aligned} \varepsilon = \frac{\left\| {\varvec{y}}(t) - \bar{{\varvec{y}}}(t) \right\| _\infty }{\left\| {\varvec{y}}(t)\right\| _\infty }. \end{aligned}$$

Here, the relative error is selected such that the accuracy of the reduced models can be compared across a range of different inputs and initial conditions whilst retaining the same relative meaning.

It is important to note that all model reduction will result in some degree of error $\varepsilon$. As a result the specific choice of reduced model to use in a given situation essentially boils down to a compromise between simplicity and accuracy. Inevitably this choice will depend upon the specific aims and context associated with the modelling work being performed, and as such it is hard to give a catch-all rule for choosing the best level of reduction to employ. In this paper we have selected the target of 5% error for our reduced linked models, and aimed to construct the minimal dimensional model that remains within this degree of error.

### Proper lumping

Proper lumping is a method of model reduction which seeks to create a lower dimensional representation of a system by partitioning the state-variables ${\varvec{x}}(t)$ into subsets, and modelling the dynamics of these subsets en masse. This is achieved via a linear operator $L: {\mathbb{R}}^n \rightarrow {\mathbb{R}}^{\hat{n}}$ that can be applied to the original state-variables, such that

5 $$\begin{aligned} \tilde{{\varvec{x}}}(t) = L{\varvec{x}}(t), \end{aligned}$$

where $\tilde{{\varvec{x}}}(t)\in {\mathbb{R}}^{\hat{n}}$ represents a reduced set of state-variables, with $\hat{n}<n$. Given such a lumping matrix *L*, a more detailed mathematical account of how to obtain the reduced, dynamical description of the model under this projection can be found in Appendix 2.

There is a range of literature describing different approaches for finding the optimal lumping matrix *L* to produce a reduced system of dimension $\hat{n}$ for a given system. Here we employ the scheme described by Dokoumetzidis and Aarons [9]. This algorithm runs an exhaustive search of possible lumping matrices to determine which produces the lowest error between simulation of the original model and the reduced model. To speed up this process, it is assumed (from justifications given in the original paper) that the lowest error *k* dimensional reduction obtained via lumping of an *n* dimensional system can also be found as the optimal lumping of two states in the $k+1$ dimensional reduction. This yields a ‘forward selection’ strategy, where 2 of the state-variables are lumped at each step, which greatly decreases the combinatorial burden of possible lumping matrices that must be evaluated.

### Balanced truncation

Balanced truncation is a method of model reduction for the simplification of systems describing an input–output type process. It is most commonly employed in the field of control theory and was originally devised in the early 1980s [31]. The method was further refined by a number of authors (e.g. [35]) and has subsequently become a well-developed one [11, 44]. Typically, it is used in the simplification of time-invariant, linear systems and seeks to remove those portions of the dynamics that contribute least to the overall input–output relationship of the model. As such it begins with systems in the form of Eq. (2) and assumes that *A* is a stable or Hurwitz matrix, such that its eigenvalues all have negative real components. This implies that the system is asymptotically stable; a property that will typically hold true for all biochemical systems.

Central to the application of balanced truncation to such a system are the concepts of observability and controllability. Broadly speaking, controllability asks to what degree the state-variables ${\varvec{x}}(t)$ of the system can be ‘moved’ or affected by the input ${\varvec{u}}(t)$. Meanwhile observability asks to what degree the state-variables of the system can be inferred or ‘observed’ from the output ${\varvec{y}}(t)$. Both concepts go some way to addressing a crucial question; to what extent and by what means does the input into the system affect its output?

To quantify these concepts requires the calculation of two matrices known as the controllability and observability Gramians (${\mathcal{P}}$ and ${\mathcal{Q}}$, respectively) for the system. Once obtained, the aim is to find a balancing transformation for the system. This is a transformation of the state-variables for which the Gramians are equalised and diagonalised and which can be achieved via use of the singular value decomposition. As a result, the state-variables produced by such a transformation are somewhat obfuscated in terms of their meaning with respect to the original model. Much like other singular value decomposition based methodologies, such as the statistical method of principle component analysis, they instead represent orthogonal directions in state-space that, when treated as the new variables of our system, describe the input–output behaviour in order of contribution. Thus the first transformed state-variable accounts for the largest contribution to the input–output relationship and each succeeding component in turn describes the most of the input–output behaviour possible under the constraint that it is orthogonal to the preceding state-variables. By omitting or ‘truncating’ the transformed state-variables that contribute least to the input–output relationship we can construct a reduced model whilst losing only relatively little accuracy.

Whilst the state-variables themselves are obscured, it is always possible to obtain an accurate description of any of the defined outputs and there is no limit on the outputs that can be defined. This is achieved via use of a generalised right inverse, that, when applied to the reduced state-variables can be used to recover approximations of the original outputs based upon the reduced system.

Such an approach has significant advantages over proper lumping; firstly, it is designed for an input–output formulation which fits well within a pharmacokinetic context. Secondly, as is shown in Dullerud and Paganini [11], an a priori error bound can be obtained for reduction under balanced truncation. A more detailed description of the mathematical steps needed to calculate and apply such a balanced truncation is given in Appendix 3.

## Results and discussion

### Reducing a PBPK model

Here we demonstrate the application of balanced truncation in reducing a PBPK model. This serves two purposes. Firstly, it allows us to reduce the model in preparation for linking with a Systems Biology type model. Secondly, it allows us to compare reduction via balanced truncation with the more commonly employed approach of proper lumping.

**Fig. 4:**
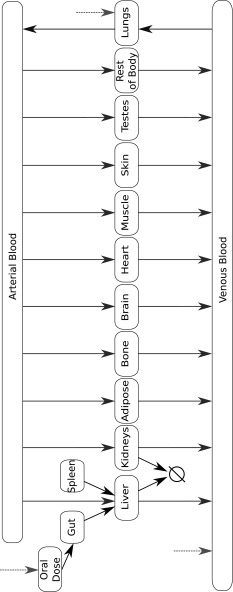
Schematic depiction of the compartments of a PBPK model for small molecule drugs due to Jones and Rowland-Yeo [17]. Here $\varnothing$ represents the drug being cleared from the body and the dotted arrows represent possible inputs into the model corresponding to routes of drug administration

A number of general frameworks for modelling PBPK have been described in the literature. Here we employ the model published by Jones and Rowland-Yeo in their recent review of PBPK modelling [17] and shown in Fig. 4. This model describes the movement of a drug between sixteen physiological compartments – adipose tissue, bone, the brain, the gut, the heart, the kidneys, the liver, the lungs, muscle tissue, the skin, the spleen, the testes, venous blood, arterial blood, an oral dosing compartment and a single compartment representing the remainder of the body. A detailed account of this PBPK model and its reduction are given in Additional file 1 - Supplementary Information.

Here we consider this general model within a control theoretic framework as earlier described by Eq. (1). When trying to understand how the administration of a particular drug propagates throughout the body, such a formulation of the problem represents a logical framework for its description. Mathematically, this model can therefore be represented by a 16 dimensional system of non-conserved, linear ODEs, which can be expressed in the form described by Eq. (2). In this case $A\in {\mathbb{R}}^{16\times 16}$ is a matrix representing the kinetic rates with which the concentration of drug moves between the compartments and ${\varvec{x}}\in {\mathbb{R}}^{16}$ represents the vector of instantaneous drug concentrations in each of the physiological compartments. Our inputs, ${\varvec{u}}(t)$, represent the times and magnitudes of the doses administered. These doses are then mapped to the compartments to which they contribute by the matrix *B*. For instance, an orally administered drug would be mapped directly to the oral dosing compartment. The outputs, ${\varvec{y}}(t)$, and their mapping *C* from the original compartment concentrations are those combinations of the compartments that the modeller seeks to predict with the system. It is possible to simply set *C* equal to the identity matrix such that all compartments are considered, however it is often the case that only some subset of the compartments (often the intravenous and effective compartments) are of clinical relevance.

Crucially to PBPK modelling, the specific values and form of the matrix *A* depends upon the model’s particular parameterisation. For models of this type, the parameters can be split into two sets.

1. The physiological parameters, found in Table 1. These parameters represent the various physiological properties of the individual to which the drug is administered. The values presented here represent a 70 kg male human with average measures of liver and kidney function, fractional tissue volumes, and blood flow from Jones and Rowland-Yeo [17].
2. The compound-specific parameters, found in Table 2. These parameters represent specific properties of the drug that has been administered. Parameterisations for three specific compounds across a reasonable pH range are given here: a strong base represented by the beta-blocker pindolol, a weak base represented by the benzodiazepine midazolam, and an acid represented by the barbiturate thiopental. Compound specific parameters were taken from Pilari and Huisinga [4]. Tissue to plasma partition coefficients were then estimated via the formulae outlined by Rodgers et al. [40, 41].

**Table 1:** Physiological parameters for the PBPK model shown in Fig. 4, their meaning, and the values used

Parameter	Meaning	Value
*BW*	Body weight (kg)	70
*QC*	Cardiac output (L/h)	$3.90\times 10^{2}$
*MPPGL*	*mg* microsomal protein per *g* liver	45
*FVad*	Fractional volume of adipose (L/kg)	$2.13\times 10^{-1}$
*FVbo*	Fractional volume of bone (L/kg)	$8.56\times 10^{-2}$
*FVbr*	Fractional volume of the brain (L/kg)	$2\times 10^{-2}$
*FVgu*	Fractional volume of the gut (L/kg)	$1.71\times 10^{-2}$
*FVhe*	Fractional volume of the heart (L/kg)	$4.7\times 10^{-3}$
*FVki*	Fractional volume of the kidneys (L/kg)	$4.4\times 10^{-3}$
*FVli*	Fractional volume of the liver (L/kg)	$2.1\times 10^{-2}$
*FVlu*	Fractional volume of the lungs (L/kg)	$7.6\times 10^{-3}$
*FVmu*	Fractional volume of muscle (L/kg)	$4\times 10^{-1}$
*FVsk*	Fractional volume of skin (L/kg)	$3.71\times 10^{-2}$
*FVsp*	Fractional volume of the spleen (L/kg)	$2.6\times 10^{-3}$
*FVte*	Fractional volume of testes (L/kg)	$1\times 10^{-2}$
*FVve*	Fractional venous volume (L/kg)	$5.14\times 10^{-2}$
*FVar*	Fractional arterial volume (L/kg)	$2.57\times 10^{-2}$
*FVpl*	Fractional volume of plasma (L/kg)	$4.24\times 10^{-2}$
*FVrb*	Fractional volume of red blood cells (L/kg)	$3.47\times 10^{-2}$
*FVre*	Fractional volume of rest of body (L/kg)	$9.98\times 10^{-2}$
*FQad*	Fractional adipose blood flow	$5\times 10^{-2}$
*FQbo*	Fractional bone blood flow	$5\times 10^{-2}$
*FQbr*	Fractional brain blood flow	$1.2\times 10^{-1}$
*FQgu*	Fractional gut blood flow	$1.46\times 10^{-1}$
*FQhe*	Fractional heart blood flow	$4\times 10^{-2}$
*FQki*	Fractional kidney blood flow	$1.9\times 10^{-1}$
*FQh*	Fractional hepatic blood flow (venous)	$2.15\times 10^{-1}$
*FQlu*	Fractional lung blood flow	1
*FQmu*	Fractional muscle blood flow	$1.7\times 10^{-1}$
*FQsk*	Fractional skin blood flow	$5\times 10^{-2}$
*FQsp*	Fractional spleen blood flow	$1.72\times 10^{-2}$

These values represent a 70 kg male human with average measures of liver and kidney function, fractional tissue volumes and blood flow. Fractional volumes represent the rough volume of each tissue proportional to overall bodyweight and the tissue specific fractional blood flows are proportional to the overall cardiac output. Parameter values are sourced from Jones and Rowland-Yeo [17]

**Table 2:** Compound specific parameters for the PBPK model shown in Fig. 4 and their meaning

Parameter	Meaning	Pindolol	Midazolam	Thiopental
*BP*	Blood to plasma ratio	0.81	0.53	0.88
${fu_{p}}$	Fraction unbound in plasma	0.41	0.05	0.18
*Ka*	Rate constant of absorption ($\mathrm{{h}}^{-1}$)	2.08	1.13	5.64
${CL_{bl}}$	Hepatic blood clearance (mL kg/min)	4.20	8.70	2.02
*Kpad*	Adipose partition coefficient	1.52	2.41	12.17
*Kpbo*	Bone partition coefficient	2.79	2.26	1.64
*Kpbr*	Brain partition coefficient	2.26	5.12	1.09
*Kpgu*	Gut partition coefficient	9.01	5.38	2.03
*Kphe*	Heart partition coefficient	8.43	2.25	1.72
*Kpki*	Kidney partition coefficient	17.94	2.51	4.85
*Kpli*	Liver partition coefficient	16.40	2.77	3.60
*Kplu*	Lung partition coefficient	14.11	3.33	1.72
*Kpmu*	Muscle partition coefficient	6.08	1.61	0.78
*Kpsk*	Skin partition coefficient	5.13	7.84	1.25
*Kpsp*	Spleen partition coefficient	11.70	1.47	0.94

Values have been given for a strong base (pindolol), a weak base (midazolam), and an acid (thiopental). Parameter values are sourced from Pilari and Huisinga [4] and tissue-to-plasma partition coefficients were approximated using the formulae of Rodgers et al. [40, 41]

We now seek to apply balanced truncation for the reduction of this system as compared with the linear, proper lumping approach as described by Pilari and Huisinga [4] which employs the algorithm originally developed by Dokoumetzidis and Aarons [9]. To test the application of balanced truncation we sought to reduce the model described above by treating the concentration of the drug in the venous compartment as the only output of interest. The dose was modelled as being orally administered in each case, with the timing and magnitude of these doses representing the model input. Given this framework, calculating reduced versions of the model via both proper lumping and balanced truncation under the given compound specific parameters yields the results given in Table 3.

**Fig. 5:**
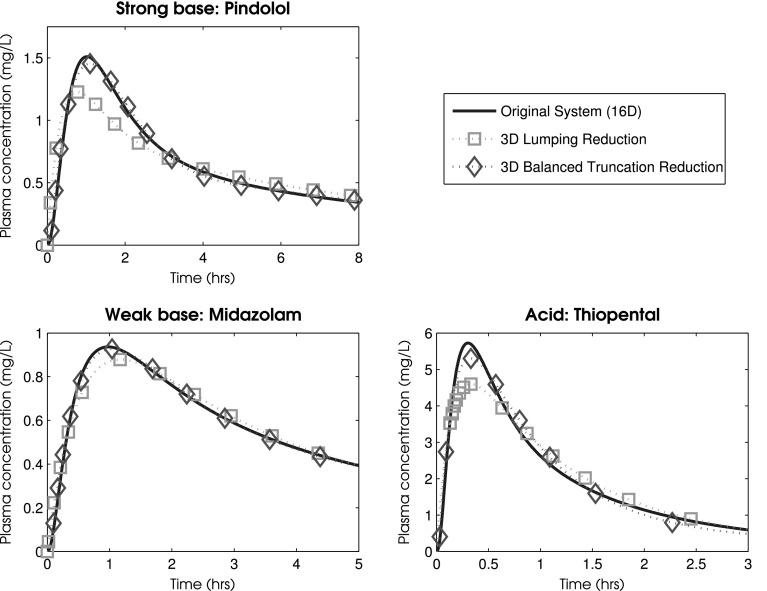
A comparison of time courses for the concentration of venous drug in the original 16 dimensional PBPK model of Fig. 4 with the 3 dimensional lumped and 3 dimensional balanced truncated reduced models. Here the oral administration of three compounds—pindolol, midazolam, and thiopental—have been simulated under the administration of a 500 mg dose. Physiological parameters were taken to represent a 70 kg male human with average measures of liver and kidney function, fractional tissue volumes and blood flow. Parameters are detailed in Tables 1 and 2

As can be seen, balanced truncation provides good reduction results even down to 3 dimensions and can often produce more accurate reductions than those obtained under lumping. Figure 5 compares the 3 dimensional reduced models obtained under balanced truncation and lumping to the original system; here, across the 3 compounds, the lumped reduction had an average maximal relative error of 19.3% whilst balanced truncation was able to attain an error of only 7.1%.

**Table 3:** Percentage maximal relative error, $\varepsilon$, associated with the reduction of the PBPK model via both lumping and balanced truncation

Dimensions	Pindolol		Midazolam		Thiopental	
	Lumping (%)	BT (%)	Lumping (%)	BT (%)	Lumping (%)	BT (%)
13	0.02	0	0.02	0	0.02	0
12	0.04	0	0.02	0	0.03	0
11	0.08	0	0.03	0	0.08	0
10	0.20	$\approx 0$	0.27	$\approx 0$	0.10	$\approx 0$
9	0.23	$\approx 0$	0.43	$\approx 0$	0.41	$\approx 0$
8	0.44	$\approx 0$	0.57	$\approx 0$	1.00	$\approx 0$
7	4.15	$\approx 0$	2.41	$\approx 0$	3.34	0.01
6	4.72	$\approx 0$	2.93	0.05	12.36	0.14
5	7.94	0.21	10.47	0.18	3.88	0.14
4	15.11	0.19	3.84	1.01	21.85	4.43
3	26.87	7.86	11.29	5.41	20.03	8.13
2	37.68	72.67	19.71	10.99	77.71	40.82
1	67.13	71.17	209.29	86.79	73.80	37.78

In each case the system is simulated under the administration of a 500 mg oral dose of the respective compound. Physiological parameters were taken to represent a 70 kg male human with average measures of liver and kidney function, fractional tissue volumes and blood flow. Here $\approx 0\%$ implies that $o(\varepsilon )<10^{-6}$

### Systems biology model reduction and linking

We now seek to demonstrate the potential application of our model linkage and reduction methodology via application to two example models. In the first instance we consider an 11 dimensional model of bacterial chemotaxis in *E. coli* - the modest scope of this model allows the application of our methods to be more easily intuited. After this we address a significantly more complex, 99 dimensional model describing the mediation of ERK activation via both the EGF and NGF receptor pathways. In both cases we employ pindolol, midazolam and thiopental as example compound specific parameterisations to represent a hypothetical drug acting on the pathways described. To demonstrate our approach, we begin by first reducing each model via lumping before linking their reduced forms with the reduced PBPK model detailed in the preceding section.

Note that the examples discussed do not relate to specific clinical cases of experimentally tested drugs, but are representative of a general case. Both examples do, however, represent receptor targets; the general methodology presented in this paper will work for any target type, but receptor signalling pathways are the most common variety of model found in the systems biology literature.

#### A model of bacterial chemotaxis

The first example is a model of chemotactic signalling in *E. coli* outlined in a 2009 paper by Tindall et al. [56]. A large body of literature exists around *E. coli* due to its popularity within experimental settings for its ease of growth and manipulation. It is a common, rod shaped, gram-negative bacteria with a large number of strains present in nature. The model discussed here pertains specifically to those strains that exhibit a chemotactic response. Chemotaxis is the process by which a cell senses an environmental chemical gradient and biases its movement towards those regions most suitable for growth and reproduction. In the model presented here, this process involves the transmembrane receptors on the surface of the bacterium sensing the local concentrations of an attractant or repellent; a decrease in attractant or an increase in repellent will cause the receptors to activate a signalling pathway inside the cell resulting in an increase of the intracellular concentration of the phosphorylated chemotactic Y protein, referred to here as $\text{CheY}_P$. This concentration, in turn, modulates the flagellum’s movement, resulting in a change of direction for the cell.

A model of bacterial chemotaxis signalling represents a good example to work with as:

1. The attractant-receptor binding mimics the typical drug-receptor binding seen in QSP modelling with the chemotactic cell response acting as the clinical endpoint;
2. Such signalling networks are typically not overly complex, but are large enough for model reduction to be warranted; and
3. Chemotaxis is well characterised in the literature, and as such models are typically fully parameterised.

Hence, it is possible to consider the external concentration of the chemotactic attractant as the input into the system and the total concentration of $\text{CheY}_P$ as the model’s output, the latter being strongly correlated with cell movement. Here we aim to create a linked reduced system bringing together a reduced version of the *E. coli* model with a reduced version of the PBPK model previously described. When the model is linked with a PBPK system, the concentration of the metabolised drug in the effective compartment is then treated as the total extracellular chemotactic attractant concentration. The full form of the chemotaxis model is given in detail in Additional file 1 - Supplementary Information.

In this instance, it was chosen that the *E. coli* cells were limited to the liver and that this represented the effective compartment. In the case of the input, it was modelled that a 150 mg dose of a chemotactic attractant was administered orally at $t=0$ with the model parameterised using those values detailed in [56]. Additionally, the PBPK model again employed the parameterisations defined by Tables 1 and 2, using pindolol, midazolam and thiopental as example compound specific parameterisations.

Reducing the PBPK model via balanced truncation, whilst seeking to preserve accuracy in the intravenous and liver compartments, allowed a reduction of the model to 3 dimensions with a maximal relative error $\varepsilon$ (in either compartment) of $15.6$, $11.4$, and 17.6% for pindolol, midazolam, and thiopental, respectively. The chemotaxis model was reduced through the application of conservation analysis, used to eliminate 4 of the system’s state-variables, followed by proper lumping under the previously described forward selection strategy. This gave the results presented in Table 4. Here, the 4 dimensional lumped model represents an excellent choice for further use in linking due to the relatively large increase in reduction error seen in going to the 3 dimensional case.

**Table 4:** Error results for the application of proper lumping to the *E. coli* chemotaxis model

Model dimensions	Lumping error (%)
6	0.15
5	0.51
4	0.54
3	4.77
2	12.88
1	75.56

The errors stated represent the maximal relative error between the outputs of original and reduced systems, relating to the total concentration of phosphorylated chemotactic protein *Y*. In order to ascertain the best lumping at a given dimensionality of reduction a wide range of attractant concentrations were tested, here however we have obtained representative error values by simulating the system under the introduction of a 10 µM concentration of attractant ligand at t = 0. This is based upon the original paper introducing the chemotaxis model [56] and the representative ligand concentration given there

**Fig. 6:**
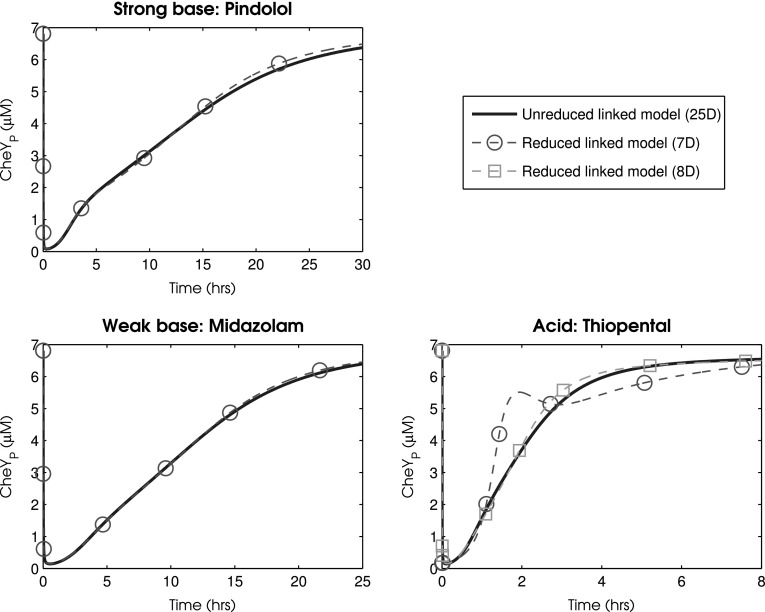
Simulated time courses for the total concentration of the phosphorylated forms of chemotactic protein CheY under the original and reduced PBPK linked chemotaxis models after oral administration of a 150 mg dose of a hypothetical chemotactic attractant. Drug specific parameters are represented by pindolol, midazolam and thiopental. Physiological parameters were taken to represent a 70 kg male human with average measures of liver and kidney function, fractional tissue volumes and blood flow. Parameters are detailed in Tables 1 and 2

As the individual methods of reduction are designed to preserve the input–output relationships of each model, the reduced PBPK and chemotaxis models can then be linked in exactly the same way as the original systems. Specifically, by setting an output of the PBPK model (the concentration of the drug in the liver) to represent the input into the chemotaxis model (which is defined as the extracellular concentration of the chemotactic attractant). Hence, linking the reduced 3 dimensional PBPK system to the reduced 4 dimensional version of the chemotaxis model yields the results given in Fig. 6. Overall, it was possible to integrate both systems whilst retaining a maximal relative error $\varepsilon$ of less than 4.6, 1.4 and 31% for the drug specific parameterisations of pindolol, midazolam and thiopental respectively. Clearly the approach provides accurate reduced models in the cases of pindolol and midazolam, but is somewhat less convincing in replicating the behaviour of thiopental; this system would require us to retain a higher number of state-variables in order to account for this drug’s faster pharmacokinetic profile. If, for example, the reduced PBPK model retains a single additional state variable (such that the PBPK model is 4 dimensional and the overall reduced model is 8 dimensional) this maximal relative error is reduced to 4.8% giving the profile also depicted in Fig. 6. Hence our methodology resulted in a reduction between the unreduced linked model and the reduced linked model from 25 state-variables to either 7 or 8 state-variables. This scale of reduction additionally yields a speed up in simulation time – through repeated simulations under Matlab’s inbuilt ode45 numerical solver (a 4th/5th order Runga-Kutta solver) a roughly 80% reduction in simulation times between the unreduced and reduced linked models was observed. For computationally intensive applications, such as parameter fitting or agent-based modelling, such a speed up in computational time provides a substantial benefit. The explicit equations for the reduced linked model are given in Additional file 1 - Supplementary Information.

#### A model of ERK activation

The second example employed here is a model of extracellular signal-regulated kinase (ERK) phosphorylation mediated via the epidermal growth factor (EGF) and the nerve growth factor (NGF) receptor pathways that was originally detailed in Sasagawa et al. [42]. This biological system commonly arises in the study of cancer and pain, and remains an area of ongoing clinical significance. This is a relatively large biochemical model describing 150 reactions and 99 species. It is also an interesting model in that it integrates two receptor pathways into one, allowing studies to be undertaken as to how they interact. Due to its size and clinical relevance, the model represents a prime candidate for the application of model reduction techniques. The SBML representation of the Sasagawa et al. model employed in this example is available at www.ebi.ac.uk/biomodels-main/BIOMD0000000049.

The total concentration of phosphorylated ERK, either in isolation or in complex, was considered as the model output. Meanwhile, EGF binding was chosen to represent the input under consideration, such that only one of the pathways described in the initial model is assumed to have a significant effect on the output of interest, therefore providing a significant opportunity for reduction. The initial condition of the system was set to be the steady-state of the network without any input. Given this framework, the model was reduced through a combination of conservation analysis and proper linear lumping. Conservation analysis was applied by finding the left-null space of the model’s associated stoichiometry matrix under the approach of QR-factorisation by Householder reflections outlined by Vallbhajosyula et al. [58]. This revealed 23 conservation relations enabling the reduction of the system to 76 state-variables. Proper lumping via the forward selection strategy as described in the methodology was then applied, yielding the results presented in Table 5.

**Table 5:** Maximal relative error $\varepsilon$ results for the reduction of the ERK activation model via proper lumping

Dimension	Lumping error (%)
75	$\approx 0$
50	0.01
25	0.52
15	1.26
14	2.21
13	2.29
12	1.21
11	3.07
10	6.02
9	10.96
8	13.12
7	14.18
6	29.53
5	39.03
4	46.47
3	54.67
2	53.52
1	55.73

In the case of the PBPK model, it was assumed that the therapeutic compartment of interest was the brain and that a dose of an antagonist that binds with the EGF receptor was administered orally at $t=0$. Once again the model parameterisation is given in Tables 1 and 2. We sought to reduce the PBPK model via balanced truncation whilst seeking to preserve accuracy in the intravenous and brain compartments. In order to maintain sufficient accuracy in the brain compartment reduction via balanced truncation required the retention of 4 state-variables in the reduced PBPK model. This yielded a maximal relative error across both the intravenous and brain compartments of 4.9, 3.8, and 9.2% in modelling the pharmacokinetics of pindolol, midazolam, and thiopental respectively.

**Fig. 7:**
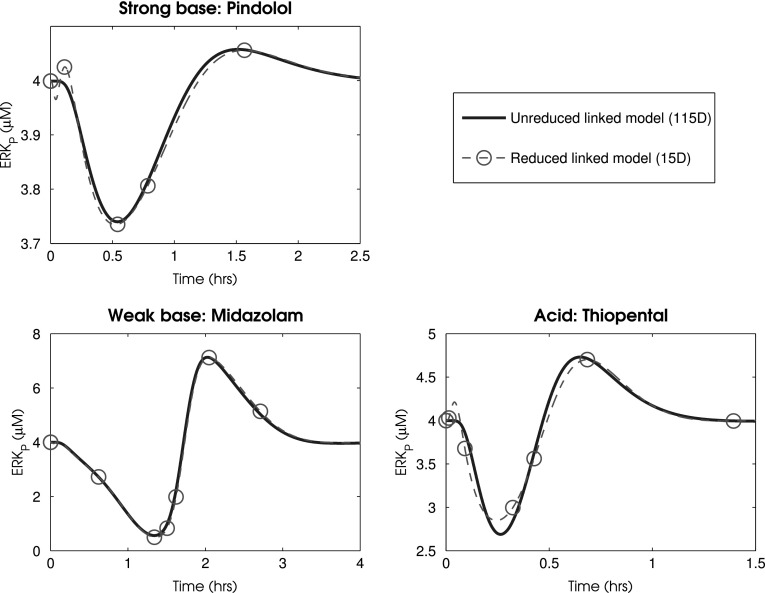
Timecourses for the total concentration of the phosphorylated forms of ERK under the original 115 dimensional and the reduced 15 dimensional PBPK linked ERK activation models after oral administration of a hypothetical EGFR antagonist. Here we simulated the system for doses of 30 mg of a hypothetical ERK antagonist represented by the drug specific parameterisations of pindolol, midazolam, and thiopental

The reduced PBPK model was then linked to the reduced ERK activation model by defining the output of the PBPK (concentration of the drug in the brain) to represent the input of the ERK activation model (the extracellular concentration of an antagonist that binds with and inhibits the EGF receptor). Employing the 11 dimensional version of the ERK-activation model and 4 dimensional PBPK model yielded the results given in Fig. 7. Here we simulated the system for doses of 30 mg of hypothetical ERK antagonists using the respective model parameterisations for pindolol, midazolam, and thiopental. This yielded a maximal relative error $\varepsilon$ of 1, 4.2, and 5.9% for pindolol, midazolam and thiopental, respectively. Overall this resulted in a reduction between the unreduced linked model and the reduced linked model from 115 state-variables to 15 and a speed up in simulation time, under Matlab’s inbuilt ode45 numerical solver, from an average of 1.764 to 0.541 s. Matlab files detailing the reduction of the ERK activation model have been made available in Additional file 2 - ERK Activation Reduction Files, use of these files requires the Matlab Symbolic package.

## Conclusions

This article has provided an account of how a model of PBPK can both be reduced and linked to systems biology type models describing target scale responses to drug administration. Doing so yields a model of enhanced pharmacodynamics or QSP that describes drug administration, metabolism, and action across multiple scales.

Crucial to the practical use of such systems is the application of model reduction methods, such as lumping and balanced truncation. Without such methods, models will typically be highly complex and intractable in the context of clinical trial data. However, as has been demonstrated throughout this paper these approaches can enable the construction of significantly simplified systems that still accurately replicate the model’s original behaviour. These models are then sufficiently simplified to be informed by and fitted against clinical trial data whilst maintaining their descriptive power across multiple scales.

This paper has focused specifically on the methods of balanced truncation and lumping for their ability to preserve input–output behaviour in reduced systems across a range of inputs. Balanced truncation was employed for the decomposed linear components of the network due to its superior properties in this regard, whilst lumping is employed for the nonlinear components. Other methods such as time-scale analysis or sensitivity analysis based approaches (outlined in much greater detail in our recent review paper [47]) could also have been considered, but their specific emphases and approaches make them less obvious choices. Throughout the paper we have sought to show how the methods might be best deployed in parallel, by aiming to decompose the overall model based upon the property of linearity and then applying each method due to its suitability based upon this criterion.

We compared the use of balanced truncation in reducing PBPK models to the more commonly applied method of proper lumping. It was demonstrated that balanced truncation can produce a more accurate reduced system than lumping. Additionally, the method is also guaranteed to produce reduced systems that reproduce the input–output behaviour not only locally to the tested inputs, but globally. As a result it represents an excellent choice for reducing the overall ‘gateway’ of the model—the point at which the main input, dosing, interacts with the system. The downside is that this approach somewhat obfuscates the meaning of the compartments in the reduced system. As a result, it is best employed in situations where a ‘black-box’ reduced system is acceptable; more specifically, it is most appropriate in situations where the structure of the reduced model is not of crucial importance, but the accuracy of its predictions as compared with the original model are.

Whilst the linked models created by the approach described in this paper have a wide range of potential benefits, there also exist a number of current limitations to their application. In particular, validation of such models is challenging; whilst clinical trials often collect data on intravenous drug concentration over time and data on the clinical endpoints observed, in vivo data on the dynamics of the subcellular species is typically not available. Additionally, mechanistically modelling how subcellular effects map to the clinical outcomes observed at the whole-body scale may require further modelling efforts. At a simpler level, even questions such as what degree of error should be tolerated in model reduction and how to select the most appropriate reduced model for a specific application remain unanswered.

Finally, the question of how best to link the intracellular and pharmacokinetic scales remains an important one. The method presented here seeks to couple the models in such a way that the outputs of one model can be treated as the inputs of the other, and vice versa. By using automated methods of reduction guaranteed to maintain the input–output behaviour across a reasonable range of inputs, this approach will give accurate reduced linked models. However, the specific form and extent of the inputs and outputs that it is necessary to define to achieve this coupling will end up limiting and defining the reduction it is possible to achieve. In short the overall reduction obtainable is likely to be improved the fewer points of coupling are required. Whilst reduction is likely to perform better the fewer inputs and outputs are defined, and the examples given in this paper possess only a single input and single output, the overall methodology remains valid for the general case of any number of defined inputs and outputs.

Irrespective of these limitations, however, the value of employing model reduction and linking in the construction of QSP models should not be understated. This approach enables us to start with pre-existing physiologically based models at multiple scales of drug activity and construct integrated, reduced models that maintain a mechanistic basis, but that are of a tractable scale. Through the use of model reduction, it is possible to shrink both the parameter space and the number of state-variables modelled. In combination with the often substantial speed-ups in simulation times observed, these approaches can make a range of computational approaches (including parameter fitting) more attainable. By selectively applying model reduction to specific portions of a network it is further possible to produce simplified systems that maintain physiological, molecular-scale detail for specific mechanisms of interest. The influence of the remainder of the system can be accounted for with a lesser degree of specificity. These directly reduced networks enable the study of specific forms of parameter variation including, for example, how patient variability at the level of protein expression might feed through to differences in dose-response. Overall, the approach outlined in this paper can be seen as providing a route to models that contain a medium level of granularity between the fully systemic level of modern approaches and the more empirical classical approaches, whilst still maintaining a physiological basis in model interpretability. By providing the tools to predict differences in patient response and consider optimal dosing strategies in a more mechanistic light, this can be seen as one stepping stone towards the ultimate goal of personalised medicine.

We feel that methods of model reduction have a vital role to play in the continuing development of QSP and that the topics discussed in this paper are fertile ground for future research. Where researchers now seek to quantitatively describe drug action in more complete terms than historical approaches allow, it is necessary that we reflect on the perennial issue of model complexity and the preservation of practical applicability.

## Electronic supplementary material

Below is the link to the electronic supplementary material.

Electronic supplementary material 1 (PDF 263 kb)

Electronic supplementary material 2 (ZIP 61 kb)

## Appendix

### Appendix 1: The Petrov–Galerkin projection

Methods of model reduction can be considered as a projection of the state-variables to a lower dimensional subspace ${\mathcal{V}}:\; \text{dim}\left( {\mathcal{V}}\right) =\hat{n}$ of the original phase-space, within which some relevant set of the system’s trajectories can be adequately approximated. Mathematically, applying such a projection to obtain a reduced dynamical system is underpinned by the Petrov–Galerkin projection which will be introduced here.

Consider a basis *B* of the subspace ${\mathcal{V}}$ such that $B= \left[ \varvec{b}_1,\ldots ,\varvec{b}_{\hat{n}}\right] \in {\mathbb{R}}^{n\times \hat{n}}$. Assuming *B* has been selected such that it provides an adequately accurate approximation of the original state-variables ${\varvec{x}}(t)$ within the subspace ${\mathcal{V}}$, then

6 $$\begin{aligned} {\varvec{x}}(t) \approx B\tilde{{\varvec{x}}}(t) \end{aligned}$$

with $\tilde{{\varvec{x}}}(t)\in {\mathbb{R}}^{\hat{n}}$ representing the reduced set of state-variables. Substituting this approximation into the original model form yields

7 $$\begin{aligned} B \frac{{\mathrm{d}}\tilde{{\varvec{x}}}}{{\mathrm{d}}t} = \varvec{f}(B\tilde{{\varvec{x}}}(t)) + \sum _{i=1}^l\varvec{g}_i(B\tilde{{\varvec{x}}}(t))u_i(t)+\varvec{\rho }(t) \end{aligned}$$

where it is assumed that *B* is time-invariant. Additionally, $\varvec{\rho }(t)\in {\mathbb{R}}^n$ is termed the residual and addresses the discrepancy emerging from the fact that $B\tilde{{\varvec{x}}}$ is typically not an exact solution of the system for all times.

Now let $\mathcal {W}$ represent a subspace that is orthogonal to the residual $\varvec{\rho }(t)$ with a basis $C\in {\mathbb{R}}^{n\times \hat{n}}$ such that $C^{\intercal }\varvec{\rho }(t)=0$ (where $\intercal$ represents the standard definition of the transpose). Hence, left multiplying Eq. (7) by $C^\intercal$ produces

8 $$\begin{aligned} C^{\intercal }B \frac{{\mathrm{d}}\tilde{{\varvec{x}}}}{{\mathrm{d}}t} = C^{\intercal }\varvec{f}(B\tilde{{\varvec{x}}}(t)) + \sum _{i=1}^lC^{\intercal }\varvec{g}_i(B\tilde{{\varvec{x}}}(t))u_i(t) \end{aligned}$$

Assuming $C^{\intercal }B$ is non-singular, this finally leads to a reduced dynamical system of the form

9 $$\begin{aligned} \frac{{\mathrm{d}}\tilde{{\varvec{x}}}}{{\mathrm{d}}t} = \left( C^{\intercal }B\right) ^{-1}C^{\intercal }\varvec{f}(B\tilde{{\varvec{x}}}(t)) + \sum _{i=1}^l \left( C^{\intercal }B\right) ^{-1}C^{\intercal }\varvec{g}_i(B\tilde{{\varvec{x}}}(t))u_i(t). \end{aligned}$$

This simplification of a dynamical system to a lower dimensional subspace is known as the Petrov–Galerkin projection. In the special case where $B=C$ it is known simply as the Galerkin projection. In that case

10 $$\begin{aligned} \left( B^{\intercal }B\right) ^{-1}B^{\intercal } = \bar{B} \end{aligned}$$

Such that $\bar{B}$ is a generalised left inverse of *B* and $\bar{B}B=I_{\hat{n}}$ (the $\hat{n}$ dimensional identity matrix).

Whilst the explanation given above provides an explanation of how to apply a Petrov–Galerkin projection, it does not provide a methodology for finding suitable bases *B* and *C* for a given model. It is methodologies of this kind that comprise the majority of the model reduction literature.

### Appendix 2: Lumping

For a general system of ODEs of the form represented by Eq. (1), a lumping is some mapping $L: {\mathbb{R}}^n \rightarrow {\mathbb{R}}^{\hat{n}}$ of the original state variables ${\varvec{x}}(t)\in {\mathbb{R}}^n$ to a reduced set $\tilde{{\varvec{x}}}(t)\in {\mathbb{R}}^{\hat{n}}$ where $\hat{n}<n$. In the case of a linear lumping this can be expressed as a projection of the form

11 $$\begin{aligned} \tilde{{\varvec{x}}}(t) = L{\varvec{x}}(t). \end{aligned}$$

In the case of a proper lumping this projection *L* becomes a matrix $L\in \left\{ 0,1\right\} ^{\hat{n}\times n}$ where each column is pairwise orthogonal, implying that each of the original state-variables corresponds to, at most, one of the lumped state-variables in the reduced model.

Given the operator *L*, the dynamics of the reduced variables $\tilde{{\varvec{x}}}(t)$ can be obtained via a Galerkin projection (see Appendix 1) applied to the original system, such that

12a $$\begin{aligned} \dot{\tilde{{\varvec{x}}}}(t)= \,& {} L\varvec{f}(\bar{L}\tilde{{\varvec{x}}}(t)) + \sum _{i=1}^l L\varvec{g}_i(\bar{L}\tilde{{\varvec{x}}}(t))u_i(t), \,\,\, \text{with} \,\,\, \tilde{{\varvec{x}}}(0)= \tilde{{\varvec{x}}}_0 = L{\varvec{x}}_0, \end{aligned}$$

12b $$\begin{aligned} \bar{{\varvec{y}}} (t)= \,& {} \varvec{h}(\bar{L}\tilde{{\varvec{x}}}(t)), \end{aligned}$$

where $\bar{L}\in {\mathbb{R}}^{n\times \hat{n}}$ represents a generalised right inverse of *L* such that $L\bar{L}=I_{\hat{n}}$, $I_{\hat{n}}$ the $\hat{n} \times \hat{n}$ identity matrix. An approximation for the original state variables from the reduced variables can be computed as

13 $$\begin{aligned} {\varvec{x}}(t) \approx \bar{L}\tilde{{\varvec{x}}}(t). \end{aligned}$$

$\bar{L}$ can be any generalised right-inverse of *L* and, as such, there exists an infinite number of ways to construct this matrix, with the specific choice affecting the error incurred by the reduced model. In this paper we follow the approach of the original lumping papers by Wei and Kuo [25, 62] which suggest selecting the generalised inverse $\bar{L}$ that reconstructs the steady-state of the system such that ${\varvec{x}}(t) = \bar{L}\tilde{{\varvec{x}}}(t)$ for $t\rightarrow \infty$. This can be constructed as

14 $$\begin{aligned} \bar{L} = XL^{\intercal }\left( LXL^{\intercal }\right) ^{-1}, \end{aligned}$$

and

$$\begin{aligned} X:=\text{diag}\left\{ {\varvec{x}}^*\right\} \end{aligned}$$

and ${\varvec{x}}^*$ represents the steady-state of the system such that $\lim _{t \rightarrow +\infty } {\varvec{x}}(t) = {\varvec{x}}^*$.

### Appendix 3: Balanced truncation

The application of balanced truncation begins with a controlled, linear system of the form described by Eq. (2). Given such a form, we then proceed by calculating two matrices known as the controllability and observability Gramians (${\mathcal{P}}$ and ${\mathcal{Q}}$, respectively) for the system. These can be obtained via solving the following Lyapunov equations,

$$\begin{aligned} A{\mathcal{P}} + {\mathcal{P}}A^{\intercal }+BB^{\intercal }&=0, \\ A^{\intercal }{\mathcal{Q}} + {\mathcal{Q}}A+C^{\intercal }C&=0. \end{aligned}$$

Balanced truncation then requires the computation the balancing transformation, which equalises and diagonalises these matrices. This can be computed in a numerically stable manner by following the proceeding steps; first, perform a Cholesky factorisation of both of the Gramians to give

$$\begin{aligned} {\mathcal{P}} = L^{\intercal }L , \,\,\text{and} \,\, {\mathcal{Q}} = R^{\intercal }R. \end{aligned}$$

Now take a singular value decomposition of the newly formed matrix $LR^{\intercal }$ to obtain

$$\begin{aligned} LR^{\intercal } = U \Sigma V^{\intercal }, \end{aligned}$$

using this, the balancing transformation *T* and its inverse $\bar{T}$ can be computed as

$$\begin{aligned} T = \Sigma ^{-\frac{1}{2}}V^{\intercal }R \,\,\text{and} \,\, \bar{T} = L^{\intercal }U\Sigma ^{-\frac{1}{2}}. \end{aligned}$$

Given a reduced dimensionality $\hat{n}$ the reduced model can be constructed via the following transformations

$$\begin{aligned} {\varvec{x}} \rightarrow {\tilde{\varvec{x}}}&= PT{\varvec{x}}, \\ A \rightarrow \tilde{A}&= PTA\bar{T}P^{\intercal },\\ B \rightarrow \tilde{B}&=PTB,\\ C \rightarrow \tilde{C}&= C\bar{T}P^{\intercal }, \end{aligned}$$

Where *P* is an $\hat{n}\times n$ matrix of the form $P = \left[ I_{\hat{n}} 0\right]$. This gives a reduced, $\hat{n}$ dimensional model of the form

$$\begin{aligned} \dot{\tilde{{\varvec{x}}}}&= \tilde{A}\tilde{{\varvec{x}}} + \tilde{B}{\varvec{u}}, \\ \bar{{\varvec{y}}}&= \tilde{C}\tilde{\varvec{x}}. \end{aligned}$$
