# Supplementary material for: Model reduction in mathematical pharmacology: Integration, reduction and linking of PBPK and systems biology models
Source: J Pharmacokinet Pharmacodyn. 2018 Mar 26;45(4):537–55. doi: 10.1007/s10928-018-9584-y (PMC6061126; doi:10.1007/s10928-018-9584-y)
Supplement: Supplementary file 1 — Electronic supplementary material 1 (PDF 263 kb) [file 10928_2018_9584_MOESM1_ESM.pdf]

Model Reduction in Mathematical Pharmacology: Integration, Reduction and Linking of PBPK and Systems Biology Models – supplementary information

Thomas J. Snowden[1,2], Piet H. van der Graaf[2,3], Marcus J. Tindall[1,4,*]
1 Department of Mathematics and Statistics, University of Reading, Reading, UK
2 Certara QSP, University of Kent Innovation Centre, CT2 7FG Canterbury, UK
3 Leiden Academic Centre for Drug Research, Universiteit Leiden, Leiden, Netherlands
4 The Institute for Cardiovascular and Metabolic Research (ICMR), University of Reading, Reading, UK
∗ **E-mail: M.Tindall@reading.ac.uk**

Contents

1 Overview of model reduction methods 1
1.1 Conservation analysis 1

2 PBPK model summary 3
2.1 Application of Balanced Truncation 4

3 Chemotaxis model summary 6
3.1 Reduction 9
3.1.1 Nondimensionalisaton 9
3.1.2 Conservation Analysis 9
3.1.3 Lumping 12

4 PBPK-Chemotaxis linked system 13

5 ERK activation model 14

1 Overview of model reduction methods

1.1 Conservation analysis

To understand the nature of conservation relations and how they might be found computationally, first note that a common means for representing the network structure underlying a system of chemical equations is that of the stoichiometry matrix. This is an $n \times m$ matrix S, with each of the rows corresponding to a single species and each of the columns to a reaction. The matrix is populated such that its entries s_{ij} give the net value of the stoichiometric coefficients (product minus reactant) of the i-th species in the j-th reaction. If the concentration of a particular species is not affected by a reaction the corresponding entry is populated with a 0. Hence, the sign of the entry indicates whether the species is a net reactant or a net product in the relevant reaction. A positive sign implies that the species is a product (i.e. the number of molecules is increased by the reaction), whilst a negative sign indicates that the species is a reactant (i.e. the number of molecules is decreased).

This matrix can be considered as mapping the vector of reaction rates $\boldsymbol{v}(\boldsymbol{x}(t))$ to the change in species concentration. Hence it is possible to represent a system of ODEs describing such chemical interactions in the form

$$\dot{\boldsymbol{x}}(t) = S\boldsymbol{v}(\boldsymbol{x}(t)). \tag{1}$$

Now turning to conservation analysis and following the ideas outlined by Reder [1], the existence of conservation relations in a model implies that

$$\Gamma\dot{\boldsymbol{x}}(t) = 0 \tag{2}$$

where Γ is an $h \times n$ matrix that will be referred to here as the conservation matrix, the rows of which represent the linear combinations of species that are constant in time. Alternatively, by integration,

$$\Gamma\boldsymbol{x}(t) = \boldsymbol{c}, \tag{3}$$

the h individual elements of which are known as conservation relations, with $\boldsymbol{c} \in \mathbb{R}^h$ representing a set of constants known as conserved values.

It is hence possible to express a model containing conservation relations in the form of a system of differential algebraic equations (DAEs). To see this, first partition $\boldsymbol{x}$ into two subsets: $\boldsymbol{x}_d$ an h dimensional subset of the species with each element corresponding to a single species involved in a given conservation relation termed the dependent species. And $\boldsymbol{x}_i$ an $n-h$ dimensional subset accommodating all remaining state-variables, termed the independent species, such that

$$\boldsymbol{x}(t) = \begin{bmatrix} \boldsymbol{x}_d(t) \\ \boldsymbol{x}_i(t) \end{bmatrix}. \tag{4}$$

Then from equation (3)

$$\Gamma \begin{bmatrix} \boldsymbol{x}_d(t) \\ \boldsymbol{x}_i(t) \end{bmatrix} = \boldsymbol{c}. \tag{5}$$

This is a system of linear equations and hence if Γ is expressed in reduced row echelon form, such that

$$\Gamma = [I_h \; N_0] \tag{6}$$

with I_h representing the h dimensional identity matrix and N_0 a $h \times (n-h)$ matrix, it becomes apparent that

$$\boldsymbol{x}_d(t) = \boldsymbol{c} - N_0\boldsymbol{x}_i(t). \tag{7}$$

This implies that the subset of dependent species $\boldsymbol{x}_d$ can be eliminated from the governing system of ODEs by substituting in the appropriate element of equation (7). Hence, given the stoichiometric form given in equation (1), a system exhibiting conservation relations can be expressed in the form of a semi-explicit system of DAEs, such that

$$\dot{\boldsymbol{x}}_i = S_i\boldsymbol{v}(\boldsymbol{x}_i(t)), \tag{8a}$$

$$\boldsymbol{x}_d(t) = N_0\boldsymbol{x}_i(t) - \boldsymbol{c}, \tag{8b}$$

where equation (8b) has been exploited in equation (8a) to obtain a system of ODEs such that state-variables $\boldsymbol{x}_d$ are no longer explicitly given. Additionally, S_i here represents the rows of the stoichiometric matrix corresponding to the independent state-variables $\boldsymbol{x}_i$.

Obtaining the conservation matrix Γ, particularly for large systems, is often not feasible from simple inspection. To understand a more algorithmic approach for obtaining this matrix, begin by recalling the stoichiometric form of a model. Decomposing the stoichiometric matrix via the same partition as the set of species leads to the system

$$\begin{pmatrix} \dot{\boldsymbol{x}}_d(t) \\ \dot{\boldsymbol{x}}_i(t) \end{pmatrix} = \begin{pmatrix} S_d \\ S_i \end{pmatrix} \boldsymbol{v}(\boldsymbol{x}_d(t), \boldsymbol{x}_i(t)). \tag{9}$$

However, via differentiation of equation (7)

$$\dot{\boldsymbol{x}}_d(t) = -N_0\dot{\boldsymbol{x}}_i(t) = -N_0S_i\boldsymbol{v}(\boldsymbol{x}_d(t), \boldsymbol{x}_i(t)). \tag{10}$$

Hence, $S_d = -N_0 S_i$ and therefore each conservation relation can be seen as corresponding to a linear dependency in the stoichiometry matrix. As such, conservation relations can be found by seeking the left null space Z_n of S (i.e. via finding the null space of S^T) such that

$$Z_n = \{ \boldsymbol{z} \in \mathbb{R}^n \,|\, S^{\intercal} \boldsymbol{z} = \boldsymbol{0} \}\,, \tag{11}$$

and hence $Z_n^{\intercal} S = \boldsymbol{0}$. This implies that

$$Z_n^{\intercal} S \boldsymbol{v}\left(\dot{\boldsymbol{x}}(t)\right) = \boldsymbol{0} = Z_n^{\intercal} \dot{\boldsymbol{x}}(t) \tag{12}$$

and therefore via comparison to equation (2) it is clear that

$$Z_n^{\intercal} = \Gamma, \tag{13}$$

such that the conservation matrix is equal to the transpose of the left null space of the stoichiometry matrix. Hence, a more mathematically rigorous approach for finding conservation relations has been provided.

For very large systems (i.e. $n > 100$) $S^{\intercal}$ will be a large and, typically, sparse matrix. As a result, solving the system of linear equations

$$S^{\intercal} \boldsymbol{z} = \boldsymbol{0} \tag{14}$$

for each conservation relation may not always be numerically stable or efficient under traditional approaches such as Gaussian elimination. Therefore, the combined algorithm employs QR factorisation via Householder reflections, as is discussed in the main text.

2 PBPK model summary

The specific PBPK modelling framework analysed in the main manuscript was originally described in Jones and Rowland-Yeo [2]. Letting $C_{Tissue}(t)$ represent the instantaneous concentration of the drug in each named tissue compartment, this framework can be used to yield the following system of ODEs

$$\frac{\mathrm{d}C_{Adipose}(t)}{\mathrm{d}t} = \frac{Q_{Adipose}\,C_{Artery}(t)}{V_{Adipose}} - \frac{Q_{Adipose}\,C_{Adipose}(t)}{V_{Adipose}\,Kp_{Adipose}}BP, \tag{15a}$$

$$\frac{\mathrm{d}C_{Bone}(t)}{\mathrm{d}t} = \frac{Q_{Bone}\,C_{Artery}(t)}{V_{Bone}} - \frac{Q_{Bone}\,C_{Bone}(t)}{V_{Bone}\,Kp_{Bone}}BP, \tag{15b}$$

$$\frac{\mathrm{d}C_{Brain}(t)}{\mathrm{d}t} = \frac{Q_{Brain}\,C_{Artery}(t)}{V_{Brain}} - \frac{Q_{Brain}\,C_{Brain}(t)}{V_{Brain}\,Kp_{Brain}}BP, \tag{15c}$$

$$\frac{\mathrm{d}C_{Gut}(t)}{\mathrm{d}t}) = \frac{Q_{Gut}\,C_{Artery}(t)}{V_{Gut}} - \frac{Q_{Gut}\,C_{Gut}(t)}{V_{Gut}\,Kp_{Gut}}BP + K_a F C_{OralDose}(t), \tag{15d}$$

$$\frac{\mathrm{d}C_{Heart}(t)}{\mathrm{d}t}) = \frac{Q_{Heart}\,C_{Artery}(t)}{V_{Heart}} - \frac{Q_{Heart}\,C_{Heart}(t)}{V_{Heart}\,Kp_{Heart}}BP, \tag{15e}$$

$$\frac{\mathrm{d}C_{Kidney}(t)}{\mathrm{d}t} = \frac{Q_{Kidney}\,C_{Artery}(t)}{V_{Kidney}} - \left(\frac{Q_{Kidney}}{Kp_{Kidney}}BP + CL_{Renal}\right)\frac{C_{Kidney}(t)}{V_{Kidney}}, \tag{15f}$$

$$\begin{aligned}\frac{\mathrm{d}C_{Liver}(t)}{\mathrm{d}t} &= \frac{Q_{Gut}\,C_{Gut}(t)}{V_{Liver}\,Kp_{Gut}}BP - \left(\frac{Q_{HepVen}}{Kp_{Liver}}BP + CL_{Int}\right)\frac{C_{Liver}(t)}{V_{Liver}} \\ &\quad + \frac{Q_{Spleen}\,C_{Spleen}(t)}{V_{Liver}\,Kp_{Spleen}}BP + \frac{Q_{HepArt}\,C_{Artery}(t)}{V_{Liver}}\end{aligned} \tag{15g}$$

$$\frac{\mathrm{d}C_{Lung}(t)}{\mathrm{d}t} = \frac{Q_{Lung}\,C_{Vein}}{V_{Lung}} - \frac{Q_{Lung}\,C_{Lung}(t)}{V_{Lung}\,Kp_{Lung}}BP, \tag{15h}$$

$$\frac{\mathrm{d}C_{Muscle}(t)}{\mathrm{d}t} = \frac{Q_{Muscle}\,C_{Artery}(t)}{V_{Muscle}} - \frac{Q_{Muscle}\,C_{Muscle}(t)}{V_{Muscle}\,Kp_{Muscle}}BP \tag{15i}$$

$$\frac{\mathrm{d}C_{Skin}(t)}{\mathrm{d}t} = \frac{Q_{Skin}\,C_{Artery}(t)}{V_{Skin}} - \frac{Q_{Skin}\,C_{Skin}(t)}{V_{Skin}\,Kp_{Skin}}BP, \tag{15j}$$

$$\frac{\mathrm{d}C_{Spleen}(t)}{\mathrm{d}t} = \frac{Q_{Spleen}\,C_{Artery}(t)}{V_{Spleen}} - \frac{Q_{Spleen}\,C_{Spleen}(t)}{V_{Spleen}\,Kp_{Spleen}}BP, \tag{15k}$$

$$\frac{\mathrm{d}C_{Testes}(t)}{\mathrm{d}t} = \frac{Q_{Testes}\,C_{Artery}(t)}{V_{Testes}} - \frac{Q_{Testes}\,C_{Testes}(t)}{V_{Testes}\,Kp_{Testes}}BP, \tag{15l}$$

$$\begin{aligned}\frac{\mathrm{d}C_{Vein}(t)}{\mathrm{d}t} &= \frac{Q_{Adipose}\,C_{Adipose}(t)}{V_{Vein}\,Kp_{Adipose}}BP + \frac{Q_{Bone}\,C_{Bone}(t)}{V_{Vein}\,Kp_{Bone}}BP \\ &+ \frac{Q_{Brain}\,C_{Brain}(t)}{V_{Vein}\,Kp_{Brain}}BP + \frac{Q_{Heart}\,C_{Heart}(t)}{V_{Vein}\,Kp_{Heart}}BP + \frac{Q_{Kidney}\,C_{Kidney}(t)}{V_{Vein}\,Kp_{Kidney}}BP \\ &+ \frac{Q_{HepVen}\,C_{Liver}(t)}{V_{Vein}\,Kp_{Liver}}BP + \frac{Q_{Muscle}\,C_{Muscle}}{V_{Vein}\,Kp_{Muscle}}BP + \frac{Q_{Skin}\,C_{Skin}(t)}{V_{Vein}\,Kp_{Skin}}BP \\ &+ \frac{Q_{Testes}\,C_{Testes}(t)}{V_{Testes}\,Kp_{Testes}}BP + \frac{Q_{RestOfBody}\,C_{RestOfBody}(t)}{V_{RestOfBody}\,Kp_{RestOfBody}}BP \\ &- \frac{Q_{Lung}\,C_{Vein}(t)}{V_{Vein}} + u_2(t),\end{aligned} \tag{15m}$$

$$\frac{\mathrm{d}C_{Artery}(t)}{\mathrm{d}t} = \frac{Q_{Lung}\,C_{Lung}(t)}{V_{Artery}\,Kp_{Lung}} - \frac{Q_{Lung}\,C_{Artery}(t)}{V_{Artery}}, \tag{15n}$$

$$\frac{\mathrm{d}C_{RestOfBody}(t)}{\mathrm{d}t} = \frac{Q_{RestOfBody}\,C_{Artery}(t)}{V_{RestOfBody}} - \frac{Q_{RestOfBody}\,C_{RestOfBody}(t)}{V_{RestOfBody}\,Kp_{RestOfBody}}BP, \tag{15o}$$

$$\frac{\mathrm{d}C_{OralDose}(t)}{\mathrm{d}t} = -K_a F C_{OralDose}(t) + u_1(t), \tag{15p}$$

where Q_{Tissue} terms represent the total blood flows associated with each of the tissues, V_{Tissue} terms describe the physiological volumes of each of the associated tissue compartments, and Kp_{Tissue} terms relate to the compound specific tissue to plasma partition coefficients. Additionally, there are two inputs into the model $u_1(t)$ and $u_2(t)$ representing routes of drug administration. $u_1(t)$ represents an oral dosing route and $u_2(t)$ represents intravenous dosing and can be defined to represent any pattern of dosing, including infusion or bolus.

Given the ODEs defined above, this 16 dimensional model is linear, and can be expressed in a control affine state-space representation of the form

$$\frac{\mathrm{d}\boldsymbol{x}(t)}{\mathrm{d}t} = A\,\boldsymbol{x}(t) + B\boldsymbol{u}(t) \tag{16}$$

where $\boldsymbol{x}(t)$ is defined as a vector representing the instantaneous tissue concentrations, such that

$$\begin{aligned}\boldsymbol{x}(t) = (&C_{Adipose}(t),\ C_{Bone}(t),\ C_{Brain}(t),\ C_{Gut}(t),\ C_{Heart}(t),\ C_{Kidney}(t),\ C_{Liver}(t),\ C_{Lung}(t),\\ &C_{Muscle}(t),\ C_{Skin}(t),\ C_{Spleen}(t),\ C_{Testes}(t),\ C_{Vein}(t),\ C_{Artery}(t),\ C_{RestOfBody}(t),\ C_{OralDose}(t))^{\mathsf{T}}\,.\end{aligned}$$

2.1 Application of Balanced Truncation

In this section we will describe how balanced truncation can be applied to yield a reduced description of the PBPK model described by the system of equations given in (15).

Balanced truncation requires that we begin with a system in the form

$$\frac{\mathrm{d}\boldsymbol{x}(t)}{\mathrm{d}t} = A\,\boldsymbol{x}(t) + B\boldsymbol{u}(t), \tag{17a}$$

$$\boldsymbol{y}(t) = C\boldsymbol{x}(t). \tag{17b}$$

From the system of equations given in (15) and both the physiological parameterisation and the drug specific parameterisations given for Midazolam in the main manuscript, we obtain the following matrix

$$A = \begin{pmatrix}
-0.29 & 0 & 0 & 0 & 0 & 0 & 0 & 0 & 0 & 0 & 0 & 0 & 10.84 & 0 \\
0 & -0.76 & 0 & 0 & 0 & 0 & 0 & 0 & 0 & 0 & 0 & 0 & 10.84 & 0 \\
0 & 0 & -3.46 & 0 & 0 & 0 & 0 & 0 & 0 & 0 & 0 & 0 & 26.01 & 0 \\
0 & 0 & 0 & -4.70 & 0 & 0 & 0 & 0 & 0 & 0 & 0 & 0 & 31.75 & 6.11 \\
0 & 0 & 0 & 0 & -11.15 & 0 & 0 & 0 & 0 & 0 & 0 & 0 & 8.67 & 0 \\
0 & 0 & 0 & 0 & 0 & -50.85 & 0 & 0 & 0 & 0 & 0 & 0 & 41.19 & 0 \\
0 & 0 & 0 & 4.70 & 0 & 0 & -10.96 & 0 & 0 & 0 & 13.29 & 0 & 11.21 & 0 \\
0 & 0 & 0 & 0 & 0 & 0 & 0 & -116.66 & 0 & 0 & 0 & 108.39 & 0 & 0 \\
0 & 0 & 0 & 0 & 0 & 0 & 0 & 0 & -0.78 & 0 & 0 & 0 & 36.85 & 0 \\
0 & 0 & 0 & 0 & 0 & 0 & 0 & 0 & 0 & -0.51 & 0 & 0 & 10.84 & 0 \\
0 & 0 & 0 & 0 & 0 & 0 & 0 & 0 & 0 & 0 & -13.29 & 0 & 3.74 & 0 \\
0.29 & 0.76 & 3.46 & 0 & 11.15 & 50.85 & 10.95 & 0 & 0.78 & 0.51 & 0 & -108.39 & 0 & 0 \\
0 & 0 & 0 & 0 & 0 & 0 & 0 & 116.66 & 0 & 0 & 0 & 0 & -216.78 & 0 \\
0 & 0 & 0 & 0 & 0 & 0 & 0 & 0 & 0 & 0 & 0 & 0 & 0 & -6.11
\end{pmatrix}$$

If we additionally define an input $u(t)$ to represent an oral dosing route and define 2 outputs, $\boldsymbol{y}(t) = (y_1(t), y_2(t))^\intercal$ representing the concentration in the gut compartment and the intravenous concentration respectively, we can additionally define the matrices B and C as

$$B = \begin{pmatrix} 0 & 0 & 0 & 0 & 0 & 0 & 0 & 0 & 0 & 0 & 0 & 0 & 0 & 1 \end{pmatrix}^\intercal,$$

and

$$C = \begin{pmatrix} 0 & 0 & 0 & 1 & 0 & 0 & 0 & 0 & 0 & 0 & 0 & 0 & 0 & 0 \\ 0 & 0 & 0 & 0 & 0 & 0 & 0 & 0 & 0 & 0 & 0 & 1 & 0 & 0 \end{pmatrix}.$$

Given this formulation, it is then possible to obtain the controllability and observability Gramians by solving the Lyapunov equations,

$$A\mathcal{P} + \mathcal{P}A^\intercal + BB^\intercal = 0,$$
$$A^\intercal \mathcal{Q} + \mathcal{Q}A + C^\intercal C = 0.$$

This yields

$$\mathcal{P} = \begin{pmatrix}
0.1159 & 0.0484 & 0.0247 & 0.0279 & 0.0025 & 0.0025 & 0.0173 & 0.0057 & 0.1617 & 0.0712 & 0.0009 & 0.0061 & 0.0031 & 0.0014 \\
0.0484 & 0.0229 & 0.0129 & 0.0169 & 0.0013 & 0.0013 & 0.0103 & 0.0030 & 0.0768 & 0.0321 & 0.0005 & 0.0032 & 0.0016 & 0.0013 \\
0.0247 & 0.0129 & 0.0088 & 0.0159 & 0.0009 & 0.0010 & 0.0093 & 0.0022 & 0.0434 & 0.0173 & 0.0003 & 0.0023 & 0.0012 & 0.0022 \\
0.0279 & 0.0169 & 0.0159 & 0.0838 & 0.0021 & 0.0024 & 0.0368 & 0.0057 & 0.0568 & 0.0210 & 0.0008 & 0.0062 & 0.0030 & 0.0486 \\
0.0025 & 0.0013 & 0.0009 & 0.0021 & 0.0001 & 0.0001 & 0.0012 & 0.0002 & 0.0044 & 0.0017 & 0.0000 & 0.0003 & 0.0001 & 0.0004 \\
0.0025 & 0.0013 & 0.0010 & 0.0024 & 0.0001 & 0.0001 & 0.0013 & 0.0003 & 0.0044 & 0.0018 & 0.0000 & 0.0003 & 0.0001 & 0.0006 \\
0.0025 & 0.0013 & 0.0010 & 0.0024 & 0.0001 & 0.0001 & 0.0013 & 0.0003 & 0.0044 & 0.0018 & 0.0000 & 0.0003 & 0.0001 & 0.0006 \\
0.0173 & 0.0103 & 0.0093 & 0.0368 & 0.0012 & 0.0013 & 0.0180 & 0.0030 & 0.0348 & 0.0130 & 0.0004 & 0.0033 & 0.0016 & 0.0141 \\
0.0057 & 0.0030 & 0.0022 & 0.0057 & 0.0002 & 0.0003 & 0.0030 & 0.0006 & 0.0101 & 0.0040 & 0.0001 & 0.0006 & 0.0003 & 0.0016 \\
0.1617 & 0.0768 & 0.0434 & 0.0568 & 0.0044 & 0.0044 & 0.0348 & 0.0101 & 0.2570 & 0.1074 & 0.0016 & 0.0108 & 0.0054 & 0.0043 \\
0.0712 & 0.0321 & 0.0173 & 0.0210 & 0.0017 & 0.0018 & 0.0130 & 0.0040 & 0.1074 & 0.0459 & 0.0006 & 0.0043 & 0.0021 & 0.0013 \\
0.0009 & 0.0005 & 0.0003 & 0.0008 & 0.0000 & 0.0000 & 0.0004 & 0.0001 & 0.0016 & 0.0006 & 0.0000 & 0.0001 & 0.0000 & 0.0002 \\
0.0061 & 0.0032 & 0.0023 & 0.0062 & 0.0003 & 0.0003 & 0.0033 & 0.0006 & 0.0108 & 0.0043 & 0.0001 & 0.0007 & 0.0003 & 0.0018 \\
0.0031 & 0.0016 & 0.0012 & 0.0030 & 0.0001 & 0.0001 & 0.0016 & 0.0003 & 0.0054 & 0.0021 & 0.0000 & 0.0003 & 0.0002 & 0.0008 \\
0.0014 & 0.0013 & 0.0022 & 0.0486 & 0.0004 & 0.0006 & 0.0141 & 0.0016 & 0.0043 & 0.0013 & 0.0002 & 0.0018 & 0.0008 & 0.0818
\end{pmatrix},$$

$$\mathcal{Q} = \begin{pmatrix}
0.0041 & 0.0045 & 0.0044 & 0.0057 & 0.0042 & 0.0041 & 0.0042 & 0.0041 & 0.0045 & 0.0044 & 0.0043 & 0.0041 & 0.0040 & 0.0066 \\
0.0045 & 0.0057 & 0.0062 & 0.0093 & 0.0060 & 0.0058 & 0.0060 & 0.0057 & 0.0057 & 0.0053 & 0.0061 & 0.0057 & 0.0056 & 0.0112 \\
0.0044 & 0.0062 & 0.0083 & 0.0175 & 0.0087 & 0.0085 & 0.0087 & 0.0081 & 0.0062 & 0.0054 & 0.0087 & 0.0083 & 0.0080 & 0.0207 \\
0.0057 & 0.0093 & 0.0175 & 0.1299 & 0.0237 & 0.0279 & 0.0236 & 0.0303 & 0.0094 & 0.0076 & 0.0199 & 0.0295 & 0.0312 & 0.0845 \\
0.0042 & 0.0060 & 0.0087 & 0.0237 & 0.0099 & 0.0102 & 0.0099 & 0.0094 & 0.0060 & 0.0052 & 0.0094 & 0.0099 & 0.0094 & 0.0255 \\
0.0041 & 0.0058 & 0.0085 & 0.0279 & 0.0102 & 0.0116 & 0.0102 & 0.0102 & 0.0058 & 0.0051 & 0.0091 & 0.0116 & 0.0102 & 0.0266 \\
0.0042 & 0.0060 & 0.0087 & 0.0236 & 0.0099 & 0.0102 & 0.0099 & 0.0094 & 0.0060 & 0.0052 & 0.0094 & 0.0099 & 0.0094 & 0.0254 \\
0.0041 & 0.0057 & 0.0081 & 0.0303 & 0.0094 & 0.0102 & 0.0094 & 0.0101 & 0.0057 & 0.0050 & 0.0087 & 0.0102 & 0.0101 & 0.0263 \\
0.0045 & 0.0057 & 0.0062 & 0.0094 & 0.0060 & 0.0058 & 0.0060 & 0.0057 & 0.0058 & 0.0053 & 0.0062 & 0.0058 & 0.0057 & 0.0113 \\
0.0044 & 0.0053 & 0.0054 & 0.0076 & 0.0052 & 0.0051 & 0.0052 & 0.0050 & 0.0053 & 0.0050 & 0.0054 & 0.0050 & 0.0050 & 0.0091 \\
0.0043 & 0.0061 & 0.0087 & 0.0199 & 0.0094 & 0.0091 & 0.0094 & 0.0087 & 0.0062 & 0.0054 & 0.0094 & 0.0088 & 0.0086 & 0.0237 \\
0.0041 & 0.0057 & 0.0083 & 0.0295 & 0.0099 & 0.0116 & 0.0099 & 0.0102 & 0.0058 & 0.0050 & 0.0088 & 0.0148 & 0.0102 & 0.0264 \\
0.0040 & 0.0056 & 0.0080 & 0.0312 & 0.0094 & 0.0102 & 0.0094 & 0.0101 & 0.0057 & 0.0050 & 0.0086 & 0.0102 & 0.0102 & 0.0261 \\
0.0066 & 0.0112 & 0.0207 & 0.0845 & 0.0255 & 0.0266 & 0.0254 & 0.0263 & 0.0113 & 0.0091 & 0.0237 & 0.0264 & 0.0261 & 0.0845
\end{pmatrix}.$$

A balancing transformation can then be found by following the detailed steps found in Appendix [???] of the main manuscript. This yields the transformation matrix for the 3 dimensional reduced case of

$$T = \begin{pmatrix} -0.0796 & -0.1139 & -0.1716 & -0.7366 & -0.2039 & -0.2195 & -0.2034 & -0.2234 & -0.1146 & -0.0992 & -0.1874 & -0.2238 & -0.2252 & -0.6099 \\ -0.1931 & -0.2093 & -0.1433 & 0.4309 & -0.0863 & -0.0594 & -0.0869 & -0.0451 & -0.2091 & -0.2091 & -0.1155 & -0.0528 & -0.0426 & 0.2096 \\ 0.0028 & 0.0507 & 0.1715 & -0.7058 & 0.1715 & 0.0848 & 0.1722 & 0.0203 & 0.0520 & 0.0252 & 0.2180 & 0.0265 & -0.0141 & 0.6425 \end{pmatrix},$$

and its inverse

$$\bar{T} = \begin{pmatrix} -0.3791 & -1.0660 & 0.1167 \\ -0.1964 & -0.4648 & -0.0201 \\ -0.1450 & -0.2180 & -0.1369 \\ -0.6079 & 0.2060 & -0.6395 \\ -0.0174 & -0.0187 & -0.0221 \\ -0.0194 & -0.0170 & -0.0256 \\ -0.2615 & -0.0030 & -0.4274 \\ -0.0458 & -0.0366 & -0.0586 \\ -0.6589 & -1.5526 & -0.0740 \\ -0.2623 & -0.6757 & 0.0253 \\ -0.0064 & -0.0066 & -0.0082 \\ -0.0498 & -0.0385 & -0.0627 \\ -0.0245 & -0.0199 & -0.0316 \\ -0.4659 & 0.4904 & 1.0269 \end{pmatrix},$$

such that the reduced 3 dimensional system can be constructed as

$$\begin{aligned} \boldsymbol{x} &\to \tilde{\boldsymbol{x}} = T\boldsymbol{x}, \\ A &\to \tilde{A} = TA\bar{T}, \\ B &\to \tilde{B} = TB, \\ C &\to \tilde{C} = C\bar{T}. \end{aligned}$$

Finally, this gives the following reduced system of differential equations

$$\frac{\mathrm{d}\tilde{x}_1(t)}{\mathrm{d}t} = -0.952\tilde{x}_1(t) + 0.454\tilde{x}_2(t) - 2.248\tilde{x}_3(t) - 0.610u(t), \tag{18a}$$
$$\frac{\mathrm{d}\tilde{x}_2(t)}{\mathrm{d}t} = 0.490\tilde{x}_1(t) - 0.312\tilde{x}_2(t) + 2.640\tilde{x}_3(t) + 0.210u(t), \tag{18b}$$
$$\frac{\mathrm{d}\tilde{x}_3(t)}{\mathrm{d}t} = 2.248\tilde{x}_1(t) - 2.699\tilde{x}_2(t) - 9.812\tilde{x}_3(t) + 0.643u(t), \tag{18c}$$

with the approximations for the outputs given as

$$C_{Gut}(t) \approx -0.608\tilde{x}_1(t) + 0.206\tilde{x}_2(t) - 0.639\tilde{x}_3(t), \tag{19a}$$
$$C_{Vein}(t) \approx -0.050\tilde{x}_1(t) - 0.039\tilde{x}_2(t) - 0.063\tilde{x}_3(t). \tag{19b}$$

3 Chemotaxis model summary

The specific model of chemotactic signalling analysed in the main text was originally described in Tindall et al. [3]. It is an 11 dimensional, nonlinear model describing a system of 12 biochemical reactions given by

$$\mathrm{CheA} \overset{k_1(L)}{\to} \mathrm{CheA}_P, \tag{20a}$$

$$\text{CheA}_P + \text{CheY} \underset{k_{-2}}{\overset{k_2}{\rightleftarrows}} \text{CheA}_P \cdot \text{CheY} \underset{k_{-7}}{\overset{k_7}{\rightleftarrows}} \text{CheA} \cdot \text{CheY}_P \underset{k_{-8}}{\overset{k_8}{\rightleftarrows}} \text{CheA} + \text{CheY}_P, \tag{20b}$$

$$\text{CheA} \cdot \text{CheY}_P \overset{k_6}{\rightarrow} \text{CheA} \cdot \text{CheY} \overset{k_{10}(L)}{\rightarrow} \text{CheA}_P \cdot \text{CheY}, \tag{20c}$$

$$\text{CheA} + \text{CheY} \underset{k_{-9}}{\overset{k_9}{\rightleftarrows}} \text{CheA} \cdot \text{CheY}, \tag{20d}$$

$$\text{CheY}_P + \text{CheZ} \underset{k_{-11}}{\overset{k_{11}}{\rightleftarrows}} \text{CheY}_P \cdot \text{CheZ} \overset{k_{12}}{\rightarrow} \text{CheY} + \text{CheZ}, \tag{20e}$$

$$\text{CheB}_P \overset{k_5}{\rightarrow} \text{CheB}, \tag{20f}$$

$$\text{CheY}_P \overset{k_6}{\rightarrow} \text{CheY}, \tag{20g}$$

$$\text{CheA}_P + \text{CheB} \overset{k_3}{\rightarrow} \text{CheA} + \text{CheB}_P. \tag{20h}$$

Whilst a more detailed account of this network can be found in the original paper, the model can be broadly understood as follows: Here, CheA represents a histidine kinase whose rate of autophosphorylation is modulated via extracellular attractant-receptor binding (in this case represented in equations (20a) and (20c) by the reaction rates $k_1(L)$ and $k_{10}(L)$, where L represents the concentration of extracellular ligand). An increase in attractant binding results in a decreased rate of phosphorylation of CheA. Two response regulator proteins (here represented by CheB and CheY and their interactions in equations (20b), (20d), and (20h)) compete to bind with protein CheA. Once bound CheA_P will transfer its phosphoryl group, hence providing the only means of phosphorylation for proteins CheB and CheY. In the case of protein CheY this phosphotransfer is also reversible via the reactions given in equation (20c). After they have been phosphorylated both response regulator proteins steadily auto-dephosphorylate as given by equations (20f) and (20g). In the case of protein CheY, however, this process can be greatly accelerated by forming a complex with the phosphatase CheZ as described by equation (20e). Note that it is the concentration of phosphorylated protein CheY that regulates the process of chemotaxis by binding with parts of the flagellar motor complex (the process is not explicitly described in this model). The model differentiates itself somewhat from previously published approaches in that explicitly describes the intermediary complexes involved in the phosphorylation of proteins CheB and CheY.

Employing the Law of Mass Action, chemical equations (20) can be modelled by the following system of ODEs

$$\frac{\mathrm{d}x_1(t)}{\mathrm{d}t} = -k_1 u(t)x_1(t) + k_3 x_2(t)x_{10}(t) + k_8 x_4(t) - k_{-8}x_1(t)x_8(t) - k_9 x_1(t)x_7(t) + k_{-9}x_5(t), \tag{21a}$$

$$\frac{\mathrm{d}x_2(t)}{\mathrm{d}t} = k_1 u(t)x_1(t) - k_2 x_2(t)x_7(t) + k_{-2}x_3(t) - k_3 x_2(t)x_{10}(t), \tag{21b}$$

$$\frac{\mathrm{d}x_3(t)}{\mathrm{d}t} = k_2 x_2(t)x_7(t) - k_{-2}x_3(t) - k_7 x_3(t) + k_{-7}x_4(t) + k_1 u(t)x_5(t), \tag{21c}$$

$$\frac{\mathrm{d}x_4(t)}{\mathrm{d}t} = -k_6 x_4(t) + k_7 x_3(t) - k_{-7}x_4(t) - k_8 x_4(t) + k_{-8}x_1(t)x_8(t), \tag{21d}$$

$$\frac{\mathrm{d}x_5(t)}{\mathrm{d}t} = k_6 x_4(t) + k_9 x_1(t)x_7(t) - k_{-9}x_5(t) - k_1 u(t)x_5(t), \tag{21e}$$

$$\frac{\mathrm{d}x_6(t)}{\mathrm{d}t} = k_{11}x_8(t)x_9(t) - k_{-11}x_6(t) - k_{12}x_6(t), \tag{21f}$$

$$\begin{aligned}\frac{\mathrm{d}x_7(t)}{\mathrm{d}t} &= -k_2 x_2(t)x_7(t) + k_{-2}x_3(t) - k_9 x_1(t)x_7(t) \\ &\quad + k_{-9}x_5(t) + k_{12}x_6(t) + k_6 x_8(t),\end{aligned} \tag{21g}$$

$$\frac{\mathrm{d}x_8(t)}{\mathrm{d}t} = k_8 x_4(t) - k_{-8}x_1(t)x_8(t) - k_{11}x_8(t)x_9(t) + k{-11}x_6(t) - k_6 x_8(t), \tag{21h}$$

Table 1. The set of parameter values associated with the chemotaxis signalling pathway model in *E. coli* as detailed in [3].

Parameter	Value	Units
k_1	3.75	s^{-1}
k_2	2.50×10^6	$(Ms)^{-1}$
k_{-2}	15	s^{-1}
k_3	1.5×10^7	$(Ms)^{-1}$
k_5	0.7	s^{-1}
k_6	8.5×10^{-2}	s^{-1}
k_7	650	s^{-1}
k_{-7}	50	s^{-1}
k_8	250	s^{-1}
k_{-8}	2.08×10^7	$(Ms)^{-1}$
k_9	7.50×10^6	$(Ms)^{-1}$
k_{-9}	15	s^{-1}
k_{10}	3.75	s^{-1}
k_{11}	5.60×10^6	$(Ms)^{-1}$
k_{-11}	0.04	s^{-1}
k_{12}	4.90	s^{-1}
A_T	7.9	μM
B_T	0.28	μM
Y_T	9.7	μM
Z_T	3.8	μM
K	2.6	μM
h	1.2	

$$\frac{\mathrm{d}x_9(t)}{\mathrm{d}t} = -k_{11}x_8(t)x_9(t) + k_{-11}x_6(t) + k_{12}x_6(t), \tag{21i}$$

$$\frac{\mathrm{d}x_{10}(t)}{\mathrm{d}t} = -k_3x_2(t)x_{10}(t) + k_5x_{11}(t), \tag{21j}$$

$$\frac{\mathrm{d}x_{11}(t)}{\mathrm{d}t} = k_3x_2(t)x_{10}(t) - k_5x_{11}(t), \tag{21k}$$

where $x_1(t) = [\text{CheA}]$, $x_2(t) = [\text{CheA}_P]$, $x_3(t) = [\text{CheA}_P \cdot \text{CheY}]$, $x_4(t) = [\text{CheA} \cdot \text{CheY}_P]$, $x_5(t) = [\text{CheA} \cdot \text{CheY}]$, $x_6(t) = [\text{CheY}_P \cdot \text{CheZ}]$, $x_7(t) = [\text{CheY}]$, $x_8(t) = [\text{CheY}_P]$, $x_9(t) = [\text{CheZ}]$, $x_{10}(t) = [\text{CheB}]$, and $x_{11}(t) = [\text{CheB}_P]$. The initial condition employed in analysing this system was

$$\begin{array}{llll} x_1(0) = 2.579\mu M, & x_2(0) = 3.084\mu M, & x_3(0) = 0.079\mu M, & x_4(0) = 0.728\mu M, \\ x_5(0) = 1.430\mu M, & x_6(0) = 2.962\mu M, & x_7(0) = 1.383\mu M, & x_8(0) = 3.118\mu M, \\ x_9(0) = 0.838\mu M, & x_{10}(0) = 0.004\mu M, & x_{11}(0) = 0.276\mu M. & \end{array}$$

The specific parameterisation employed for this model can be found in Table 1.

To understand the input u, note that receptor binding of the extracellular chemotactic ligand (denoted by L) is described by Michealis-Menten kinetics, such that

$$k_1(L) = k_{10}(L) = k_1\left(1 - \frac{L^h}{K^h + L^h}\right), \tag{22}$$

where K is a Michaelis-Menten constant and h is a Hill-coefficient. For the sake of simplicity the input $u(t)$ is thus defined here to be

$$u(t) = 1 - \frac{L^h}{K^h + L^h} \tag{23}$$

such that $k_1(L) = k_{10}(L) = k_1 u(t)$.

3.1 Reduction

In the following sections we will describe how the model of bacterial chemotaxis can be reduced via nondimensionalisation, conservation analysis and lumping to yield the results given in the main manuscript.

3.1.1 Nondimensionalisaton

We began by sampling a range of possible nondimensionalisations to obtain a representation of the system with the best numerical properties. This led to equations (24) with the associated parameters from Table 2,

$$\frac{\mathrm{d}z_1(\tau)}{\mathrm{d}\tau} = -\alpha_1 z_1(\tau)u(\tau) + \alpha_4 z_2(\tau)z_{10}(\tau) + z_4(\tau) - \alpha_9 z_1(\tau)z_8(\tau) - \alpha_{10} z_1(\tau)z_7(\tau) + \alpha_{11} z_5(\tau), \tag{24a}$$

$$\frac{\mathrm{d}z_2(\tau)}{\mathrm{d}\tau} = \alpha_1 z_1(\tau)u(\tau) - \alpha_2 z_2(\tau)z_7(\tau) + \alpha_3 z_3(\tau) - \alpha_4 z_2(\tau)z_{10}(\tau), \tag{24b}$$

$$\frac{\mathrm{d}z_3(\tau)}{\mathrm{d}\tau} = \alpha_2 z_2(\tau)z_7(\tau) - \alpha_3 z_3(\tau) - \alpha_7 z_3(\tau) + \alpha_8 z_4(\tau) + \alpha_1 z_5(\tau)u(\tau), \tag{24c}$$

$$\frac{\mathrm{d}z_4(\tau)}{\mathrm{d}\tau} = -\alpha_6 z_4(\tau) + \alpha_7 z_3(\tau) - \alpha_8 z_4(\tau) - z_4(\tau) + \alpha_9 z_1(\tau)z_8(\tau), \tag{24d}$$

$$\frac{\mathrm{d}z_5(\tau)}{\mathrm{d}\tau} = \alpha_6 z_4(\tau) + \alpha_{10} z_1(\tau)z_7(\tau) - \alpha_{11} z_5(\tau) - \alpha_1 z_5(\tau)u(\tau), \tag{24e}$$

$$\frac{\mathrm{d}z_6(\tau)}{\mathrm{d}\tau} = \alpha_{13} z_8(\tau)z_9(\tau) - \alpha_{14} z_6(\tau) - \alpha_{16} z_6(\tau), \tag{24f}$$

$$\frac{\mathrm{d}z_7(\tau)}{\mathrm{d}\tau} = -\alpha_2 z_2(\tau)z_7(\tau) + \alpha_3 z_3(\tau) - \alpha_{10} z_1(\tau)z_7(\tau) + \alpha_{11} z_5(\tau) + \alpha_{17} z_6(\tau) + \alpha_6 z_8(\tau), \tag{24g}$$

$$\frac{\mathrm{d}z_8(\tau)}{\mathrm{d}\tau} = z_4(\tau) - \alpha_9 z_1(\tau)z_8(\tau) - \alpha_{12} z_8(\tau)z_9(\tau) + \alpha_{15} z_6(\tau) - \alpha_6 z_8(\tau), \tag{24h}$$

$$\frac{\mathrm{d}z_9(\tau)}{\mathrm{d}\tau} = -\alpha_{13} z_8(\tau)z_9(\tau) + \alpha_{14} z_6(\tau) + \alpha_{16} z_6(\tau), \tag{24i}$$

$$\frac{\mathrm{d}z_{10}(\tau)}{\mathrm{d}\tau} = -\alpha_4 z_2(\tau)z_{10}(\tau) + \alpha_5 z_{11}(\tau), \tag{24j}$$

$$\frac{\mathrm{d}z_{11}(\tau)}{\mathrm{d}\tau} = \alpha_4 z_2(\tau)z_{10}(\tau) - \alpha_5 z_{11}(\tau). \tag{24k}$$

Where $t = k_8\tau$, $x_1(t) = B_T z_1(\tau)$, $x_2(t) = B_T z_2(\tau)$, $x_3(t) = B_T z_3(\tau)$, $x_4(t) = B_T z_4(\tau)$, $x_5(t) = B_T z_5(\tau)$, $x_6(t) = A_T z_6(\tau)$, $x_7(t) = B_T z_7(\tau)$, $x_8(t) = B_T z_8(\tau)$, $x_9(t) = A_T z_9(\tau)$, $x_{10}(t) = B_T z_{10}(\tau)$, and $x_{11}(t) = B_T z_{11}(\tau)$.

3.1.2 Conservation Analysis

Now we select species CheA, CheA $\cdot$ CheY, CheZ, and CheB_P for algebraic replacement via the application of conservation relations.This can be achieved by substituting the following algebraic equations into the set

Table 2. The set of nondimensionalised parameter values associated with the chemotaxis signalling pathway model in *E. coli* as defined by equation (24).

Parameter	Original Expression	Value
α_1	$\frac{k_1}{k_8}$	0.0150
α_2	$\frac{k_2 B_T}{k_8}$	0.0028
α_3	$\frac{k_{-2}}{k_8}$	0.06
α_4	$\frac{k_3 B_T}{k_8}$	0.0168
α_5	$\frac{k_5}{k_8}$	0.0028
α_6	$\frac{k_6}{k_8}$	3.4×10^{-4}
α_7	$\frac{k_7}{k_8}$	2.6
α_8	$\frac{k_{-7}}{k_8}$	0.2
α_9	$\frac{k_{-8} B_T}{k_8}$	0.0233
α_{10}	$\frac{k_9 B_T}{k_8}$	0.0084
α_{11}	$\frac{k_{-9}}{k_8}$	0.06
α_{12}	$\frac{k_{11} A_T}{k_8}$	0.1770
α_{13}	$\frac{k_{11} B_T}{k_8}$	0.0063
α_{14}	$\frac{k_{-11}}{k_8}$	1.6×10^{-4}
α_{15}	$\frac{k_{-11} A_T}{k_8 B_T}$	0.0045
α_{16}	$\frac{k_{12}}{k_8}$	0.0196
α_{17}	$\frac{k_{12} A_T}{k_8 B_T}$	0.5530

Table 3. An additional set of nondimensionalised parameter values associated with the chemotaxis signalling pathway model in *E. coli* as defined by equation (29).

Parameter	Original Expression	Value
β_1	$\frac{A_T}{B_T}$	28.21
β_2	$\frac{Y_T}{B_T}$	34.64
β_3	$\frac{Z_T}{B_T}$	13.57
β_4	$\frac{Z_T}{A_T}$	0.48

of nondimensionalised differential equations represented by equations (24),

$$[\text{CheB}_P] = B_T - [\text{CheB}] \tag{25}$$
$$[\text{CheZ}] = Z_T - [\text{CheY}_P \cdot \text{CheZ}] \tag{26}$$
$$[\text{CheA} \cdot \text{CheY}] = Y_T - [\text{CheY}] - [\text{CheY}_P] - [\text{CheA}_P \cdot \text{CheY}] - [\text{CheA} \cdot \text{CheY}_P] - [\text{CheY}_P \cdot \text{CheZ}] \tag{27}$$
$$[\text{CheA}] = A_T - Y_T + [\text{CheY}_P] + [\text{CheY}] + [\text{CheY}_P \cdot \text{CheZ}] - [\text{CheA}_P] \tag{28}$$

In the nondimensionalised form, this yields the following system

$$\frac{\mathrm{d}z_2(\tau)}{\mathrm{d}\tau} =(z_7(\tau) + z_8(\tau) - z_2(\tau) + \beta_1 z_6(\tau) + \beta_1 - \beta_2)\alpha_1 u(\tau) - \alpha_2 z_2(\tau) z_7(\tau) + \alpha_3 z_3(\tau) - \alpha_4 z_2(\tau) z_{10}(\tau), \tag{29a}$$
$$\frac{\mathrm{d}z_3(\tau)}{\mathrm{d}\tau} =(\beta_2 - z_7(\tau) - z_3(\tau) - z_4(\tau) - \beta_1 z_6(\tau) - z_8(\tau))\alpha_1 u(\tau) + \alpha_2 z_2(\tau) z_7(\tau) - \alpha_3 z_3(\tau) - \alpha_7 z_3(\tau) + \alpha_8 z_4(\tau), \tag{29b}$$
$$\frac{\mathrm{d}z_4(\tau)}{\mathrm{d}\tau} =-\alpha_6 z_4(\tau) + \alpha_7 z_3(\tau) - \alpha_8 z_4(\tau) - z_4(\tau) + (z_7(\tau) + z_8(\tau) - z_2(\tau) + \beta_1 z_6(\tau) + \beta_1 - \beta_2)\alpha_9 z_8(\tau), \tag{29c}$$
$$\frac{\mathrm{d}z_6(\tau)}{\mathrm{d}\tau} =(\beta_3 - z_6(\tau))\alpha_{13} z_8(\tau) - \alpha_{14} z_6(\tau) - \alpha_{16} z_6(\tau), \tag{29d}$$
$$\frac{\mathrm{d}z_7(\tau)}{\mathrm{d}\tau} =-\alpha_2 z_2(\tau) z_7(\tau) + \alpha_3 z_3(\tau) + \alpha_{17} z_6(\tau) + \alpha_6 z_8(\tau) - (z_7(\tau) + z_8(\tau) - z_2(\tau) + \beta_1 z_6(\tau) + \beta_1 - \beta_2)\alpha_{10} z_7(\tau) + \alpha_{11}(\beta_2 - z_7(\tau) - z_3(\tau) - z_4(\tau) - \beta_1 z_6(\tau) - z_8(\tau)), \tag{29e}$$
$$\frac{\mathrm{d}z_8(\tau)}{\mathrm{d}\tau} =z_4(\tau) + \alpha_{15} z_6(\tau) - \alpha_6 z_8(\tau) + (z_7(\tau) + z_8(\tau) - z_2(\tau) + \beta_1 z_6(\tau) + \beta_1 - \beta_2)\alpha_9 z_8(\tau) - (z_6(\tau) - \beta_4)\alpha_{12} z_8(\tau), \tag{29f}$$
$$\frac{\mathrm{d}z_{10}(\tau)}{\mathrm{d}\tau} =-\alpha_4 z_2(\tau) z_{10}(\tau) - \alpha_5 z_{10}(\tau) + \alpha_5, \tag{29g}$$

(29h)

with the additional nondimensional parameters given in Table 3

3.1.3 Lumping

We then proceed to reduce the system via proper lumping to a 4 dimensional form. In this section we will symbolically reproduce this reduced model. Through the forward selection procedure described in the main text, the algorithm determines that the optimal scheme is to lump together state-variables $z_3(\tau)$, $z_4(\tau)$, and $z_8(\tau)$ as one lumped variable, and $z_7(\tau)$ and $z_{10}(\tau)$. These lumped variables correspond to the original species $\text{CheA}_P \cdot \text{CheY}$, $\text{CheA} \cdot \text{CheY}_P$, CheY_P, and CheY, CheB, respectively.

If we assume a vector of state-variables, such that $\boldsymbol{y}(\tau) = [y_2(\tau), y_3(\tau), y_4(\tau), y_6(\tau), y_7(\tau), y_8(\tau), y_{10}(\tau)]^\intercal$, then this scheme corresponds to the lumping matrix

$$L = \begin{bmatrix} 1 & 0 & 0 & 0 & 0 & 0 & 0 \\ 0 & 1 & 1 & 0 & 0 & 1 & 0 \\ 0 & 0 & 0 & 1 & 0 & 0 & 0 \\ 0 & 0 & 0 & 0 & 1 & 0 & 1 \end{bmatrix}.$$

Hence we have a set of reduced state-variables $\tilde{\boldsymbol{x}}(\tau) = L\boldsymbol{z}(\tau)$ such that

$$\begin{bmatrix} \tilde{x}_1(\tau) \\ \tilde{x}_2(\tau) \\ \tilde{x}_3(\tau) \\ \tilde{x}_4(\tau) \end{bmatrix} = \begin{bmatrix} z_2(\tau) \\ z_3(\tau) + z_4(\tau) + z_8(\tau) \\ z_5(\tau) \\ z_7(\tau) + z_{10}(\tau) \end{bmatrix}$$

Following the general descriptions provided in the main text it is now necessary to construct a generalised right inverse $\bar{L}$ of the lumping matrix prior to application of the Galerkin projection. Such a matrix can be constructed by computing

$$\bar{L} = XL^\intercal \left(LXL^\intercal\right)^{-1}, \tag{30}$$

where X represents a diagonal matrix, typically containing steady-state values of the system. In the case of the nondimensionalised model described above, this would yield a matrix of the form

$$\bar{L} = \begin{bmatrix} 1 & 0 & 0 & 0 \\ 0 & \frac{z_3^*}{z_3^*+z_4^*+z_8^*} & 0 & 0 \\ 0 & \frac{z_4^*}{z_3^*+z_4^*+z_8^*} & 0 & 0 \\ 0 & 0 & 1 & 0 \\ 0 & 0 & 0 & \frac{z_7^*}{z_7^*+z_{10}^*} \\ 0 & \frac{z_8^*}{z_3^*+z_4^*+z_8^*} & 0 & 0 \\ 0 & 0 & 0 & \frac{z_{10}^*}{z_7^*+z_{10}^*} \end{bmatrix}.$$

Given these matrices it is then possible to construct a reduced description of the system's dynamical behaviour via the Galerkin projection, this yields the analytically reduced system

$$\begin{aligned} \frac{\mathrm{d}\tilde{x}_1(\tau)}{\mathrm{d}\tau} &= \left(\beta_1\tilde{x}_3(\tau) + \gamma_3\tilde{x}_2(\tau) + \gamma_4\tilde{x}_4(\tau) + \beta_1 - \beta_2 - \tilde{x}_1(\tau)\right)\alpha_1 u(t) - \alpha_2\tilde{x}_1(\tau)\gamma_4\tilde{x}_4(\tau) \\ &\quad + \alpha_3\gamma_1\tilde{x}_2(\tau) - \alpha_4\tilde{x}_1(\tau)\gamma_5\tilde{x}_4(\tau) \end{aligned} \tag{31a}$$

Table 4. An additional set of parameter values associated with the lumped chemotaxis signalling pathway model in *E. coli* as defined by equation (31).

Parameter	Expression	Values Used
γ_1	$\frac{z_3^*}{z_3^*+z_4^*+z_8^*}$	0.0201
γ_2	$\frac{z_4^*}{z_3^*+z_4^*+z_8^*}$	0.1855
γ_3	$\frac{z_8^*}{z_3^*+z_4^*+z_8^*}$	0.7944
γ_4	$\frac{z_7^*}{z_7^*+z_{10}^*}$	0.9986
γ_5	$\frac{z_{10}^*}{z_7^*+z_{10}^*}$	0.0014

$$
\begin{aligned}
\frac{\mathrm{d}\tilde{x}_2(\tau)}{\mathrm{d}\tau} &= \left(-\beta_1\tilde{x}_3(\tau) - \gamma_1\tilde{x}_2(\tau) - \gamma_2\tilde{x}_2(\tau) - \gamma_3\tilde{x}_2(\tau) - \gamma_4\tilde{x}_4(\tau) + \beta_2\right)\alpha_1 u(t) + \alpha_2\tilde{x}_1(\tau)\gamma_4\tilde{x}_4(\tau) \\
&\quad - \alpha_3\gamma_1\tilde{x}_2(\tau) + \alpha_6\gamma_2\tilde{x}_2(\tau) + 2\left(\beta_1\tilde{x}_3(\tau) + \gamma_3\tilde{x}_2(\tau) + \gamma_4\tilde{x}_4(\tau) + \beta_1 - \beta_2 - \tilde{x}_1(\tau)\right)\alpha_9\gamma_3\tilde{x}_2(\tau) \\
&\quad + \alpha_{15}\tilde{x}_3(\tau) - \alpha_6\gamma_3\tilde{x}_2(\tau) - \left(\tilde{x}_3(\tau) - \beta_4\right)\alpha_{12}\gamma_3\tilde{x}_2(\tau) \qquad (31b) \\
\frac{\mathrm{d}\tilde{x}_3(\tau)}{\mathrm{d}\tau} &= \left(\beta_3 - \tilde{x}_3(\tau)\right)\alpha_{13}\gamma_3\tilde{x}_2(\tau) - \alpha_{14}\tilde{x}_3(\tau) - \alpha_{16}\tilde{x}_3(\tau) \qquad (31c) \\
\frac{\mathrm{d}\tilde{x}_4(\tau)}{\mathrm{d}\tau} &= -\alpha_2\tilde{x}_1(\tau)\gamma_4\tilde{x}_4(\tau) + \alpha_3\gamma_1\tilde{x}_2(\tau) + \alpha_17\tilde{x}_3(\tau) + \alpha_6\gamma_3\tilde{x}_2(\tau) \\
&\quad - \left(\beta_1\tilde{x}_3(\tau) + \gamma_3\tilde{x}_2(\tau) + \gamma_4\tilde{x}_4(\tau) + \beta_1 - \beta_2 - \tilde{x}_1(\tau)\right)\alpha_{10}\gamma_4\tilde{x}_4(\tau) \\
&\quad + \alpha_{11}\left(-\beta_1\tilde{x}_3(\tau) - \gamma_1\tilde{x}_2(\tau) - \gamma_2\tilde{x}_2(\tau) - \gamma_3\tilde{x}_2(\tau) - \gamma_4\tilde{x}_4(\tau) + \beta_2\right) \\
&\quad - \alpha_4\tilde{x}_1(\tau)\gamma_5\tilde{x}_4(\tau) - \alpha_5\gamma_5\tilde{x}_4(\tau) + \alpha_5 \qquad (31d)
\end{aligned}
$$

where the new parameters (γ_1, γ_2, γ_3, γ_4, and γ_5) are defined in Table 4.

4 PBPK-Chemotaxis linked system

Given the reduced PBPK model represented by the systems of equations (18) and (19) and the reduced chemotaxis systems biology model represented by equations (31) it is now possible to construct a reduced linked QSP model as described in the main manuscript. This is achieved by allowing the predicted concentration of the drug in the gut compartment to feed into the systems biology model to represent the concentration of the chemotactic ligand L as described in equation (23). Doing so yields the following 7 dimensional reduced linked system

$$
\begin{aligned}
\frac{\mathrm{d}\tilde{x}_1(\tau)}{\mathrm{d}\tau} &= \frac{1}{k_8}\left(-0.952\tilde{x}_1(\tau) + 0.454\tilde{x}_2(\tau) - 2.248\tilde{x}_3(\tau) - 0.610D(\tau)\right), \qquad (32a) \\
\frac{\mathrm{d}\tilde{x}_2(\tau)}{\mathrm{d}\tau} &= \frac{1}{k_8}\left(0.490\tilde{x}_1(\tau) - 0.312\tilde{x}_2(\tau) + 2.640\tilde{x}_3(\tau) + 0.210D(\tau)\right), \qquad (32b) \\
\frac{\mathrm{d}\tilde{x}_3(\tau)}{\mathrm{d}\tau} &= \frac{1}{k_8}\left(2.248\tilde{x}_1(\tau) - 2.699\tilde{x}_2(\tau) - 9.812\tilde{x}_3(\tau) + 0.643D(\tau)\right), \qquad (32c) \\
\frac{\mathrm{d}\tilde{x}_4(\tau)}{\mathrm{d}\tau} &= \left(\beta_1\tilde{x}_6(\tau) + \gamma_3\tilde{x}_5(\tau) + \gamma_4\tilde{x}_7(\tau) + \beta_1 - \beta_2 - \tilde{x}_4(\tau)\right)\alpha_1 u(t) - \alpha_2\tilde{x}_4(\tau)\gamma_4\tilde{x}_7(\tau) \\
&\quad + \alpha_3\gamma_1\tilde{x}_5(\tau) - \alpha_4\tilde{x}_4(\tau)\gamma_5\tilde{x}_7(\tau) \qquad (32d)
\end{aligned}
$$

$$\begin{aligned}
\frac{\mathrm{d}\tilde{x}_5(\tau)}{\mathrm{d}\tau} &= \left(-\beta_1\tilde{x}_6(\tau) - \gamma_1\tilde{x}_5(\tau) - \gamma_2\tilde{x}_5(\tau) - \gamma_3\tilde{x}_5(\tau) - \gamma_4\tilde{x}_7(\tau) + \beta_2\right)\alpha_1 u(t) + \alpha_2\tilde{x}_4(\tau)\gamma_4\tilde{x}_7(\tau) \\
&\quad - \alpha_3\gamma_1\tilde{x}_5(\tau) + \alpha_6\gamma_2\tilde{x}_5(\tau) + 2\left(\beta_1\tilde{x}_6(\tau) + \gamma_3\tilde{x}_5(\tau) + \gamma_4\tilde{x}_7(\tau) + \beta_1 - \beta_2 - \tilde{x}_4(\tau)\right)\alpha_9\gamma_3\tilde{x}_5(\tau) \\
&\quad + \alpha_{15}\tilde{x}_6(\tau) - \alpha_6\gamma_3\tilde{x}_5(\tau) - \left(\tilde{x}_6(\tau) - \beta_4\right)\alpha_{12}\gamma_3\tilde{x}_5(\tau) \qquad (32e)\\
\frac{\mathrm{d}\tilde{x}_6(\tau)}{\mathrm{d}\tau} &= \left(\beta_3 - \tilde{x}_6(\tau)\right)\alpha_{13}\gamma_3\tilde{x}_5(\tau) - \alpha_{14}\tilde{x}_6(\tau) - \alpha_{16}\tilde{x}_6(\tau) \qquad (32f)\\
\frac{\mathrm{d}\tilde{x}_7(\tau)}{\mathrm{d}\tau} &= -\alpha_2\tilde{x}_4(\tau)\gamma_4\tilde{x}_7(\tau) + \alpha_3\gamma_1\tilde{x}_5(\tau) + \alpha_17\tilde{x}_6(\tau) + \alpha_6\gamma_3\tilde{x}_5(\tau) \\
&\quad - \left(\beta_1\tilde{x}_6(\tau) + \gamma_3\tilde{x}_5(\tau) + \gamma_4\tilde{x}_7(\tau) + \beta_1 - \beta_2 - \tilde{x}_4(\tau)\right)\alpha_{10}\gamma_4\tilde{x}_7(\tau) \\
&\quad + \alpha_{11}\left(-\beta_1\tilde{x}_6(\tau) - \gamma_1\tilde{x}_5(\tau) - \gamma_2\tilde{x}_5(\tau) - \gamma_3\tilde{x}_5(\tau) - \gamma_4\tilde{x}_7(\tau) + \beta_2\right) \\
&\quad - \alpha_4\tilde{x}_4(\tau)\gamma_5\tilde{x}_7(\tau) - \alpha_5\gamma_5\tilde{x}_7(\tau) + \alpha_5 \qquad (32g)
\end{aligned}$$

where we have

$$u(t) = 1 - \frac{\left(-0.608\tilde{x}_1(t) + 0.206\tilde{x}_2(t) - 0.639\tilde{x}_3(t)\right)^h}{K^h + \left(-0.608\tilde{x}_1(t) + 0.206\tilde{x}_2(t) - 0.639\tilde{x}_3(t)\right)^h} \qquad (33)$$

and $D(t)$ represents the time-course of an orally administered dosing regimen.

5 ERK activation model

An SBML formatted version of the ERK activation can be obtained at at `www.ebi.ac.uk/biomodels-main/BIOMD0000000049`. Many models such as this can commonly be obtained from online model repositories stored in the form of Systems Biology Markup Language (SBML) — a standardised format for the representation, storage and easy communication of Systems Biology models [4]. Publicly open databases, such as the BioModels Database, contain thousands of such models enabling researchers to share their work in a more accessible way.

The ERK activation model, outlined in Sasagawa et al. [5], describes extracellular signal-regulated kinase (ERK) activation as mediated via the epidermal growth factor (EGF) and the nerve growth factor (NGF) receptor pathways. It features 99 chemical species and 150 reactions. Due to the size and complexity of the ERK activation model it is not practical to provide the same level of detail into its reduction as was provided for the model of bacterial chemotaxis signalling and the PBPK model. The general procedure We have, however, provided a number of Matlab files that describe the form of the model at various stages of reduction along with its initial conditions, associated lumping matrices and their generalised inverses. Use of these files does require Matlab's Symbolic Math toolbox. All of this is made available in Additional File [???].

References

[1] Reder, C.: Metabolic control theory: a structural approach. Journal of Theoretical Biology **135**(2), 175–201 (1988)

[2] Jones, H., Rowland-Yeo, K.: Basic concepts in physiologically based pharmacokinetic modeling in drug discovery and development. CPT: Pharmacometrics & Systems Pharmacology **2**(8), 63 (2013)

[3] Tindall, M., Porter, S., Wadhams, G., Maini, P., Armitage, J.: Spatiotemporal modelling of CheY complexes in *Escherichia coli* chemotaxis. Progress in Biophysics and Molecular Biology **100**(1), 40–46 (2009)

[4] Hucka, M., Finney, A., Sauro, H.M., Bolouri, H., Doyle, J.C., Kitano, H., Arkin, A.P., Bornstein, B.J., Bray, D., Cornish-Bowden, A., *et al.*: The systems biology markup language (SBML): a medium for representation and exchange of biochemical network models. Bioinformatics **19**(4), 524–531 (2003)

[5] Sasagawa, S., Ozaki, Y.-i., Fujita, K., Kuroda, S.: Prediction and validation of the distinct dynamics of transient and sustained ERK activation. Nature Cell Biology **7**(4), 365–373 (2005)
